# Supplementary material for: Deciphering immune predictors of immunotherapy response: A multiomics approach at the pan-cancer level
Source: Cell Rep Med. 2025 Mar 6;6(4):101992. doi: 10.1016/j.xcrm.2025.101992 (PMC12047473; doi:10.1016/j.xcrm.2025.101992)
Supplement: Document S1. Figures S1–S5 [file mmc1.pdf]

Cell Reports Medicine, Volume *6*

Supplemental information

Deciphering immune predictors of immunotherapy response: A multiomics approach at the pan-cancer level

Xuexin Li, Lu Pan, Weiyuan Li, Bingyang Liu, Chunjie Xiao, Valerie Chew, Xuan Zhang, Wang Long, Florent Ginhoux, Joseph Loscalzo, Marcus Buggert, Xiaolu Zhang, Ren Sheng, and Zhenning Wang

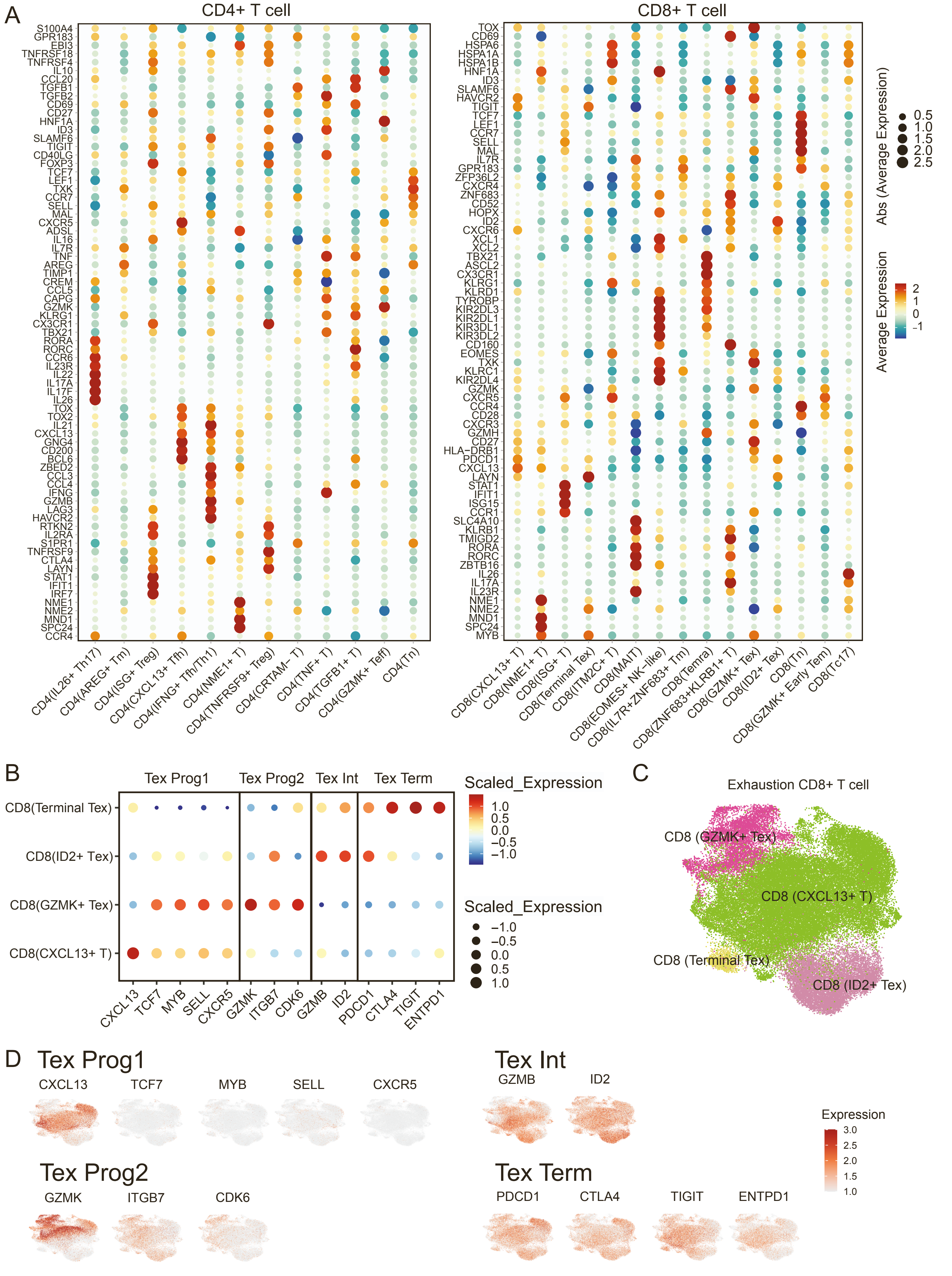

Figure S1. Expressions of signature genes in CD4 and CD8 T cells. (A) Signature genes for all the CD4 and CD8 T cells. Size of dots represented percentage of cells expressing the genes, and color gradient indicated average expression of the genes. (B) Signature genes for the four exhaustive CD8 T cell types. (C) UMAP representation of the four exhaustive CD8 T cell types and (D) the average gene expression of the signature genes across these cells. Related to Figure 2.

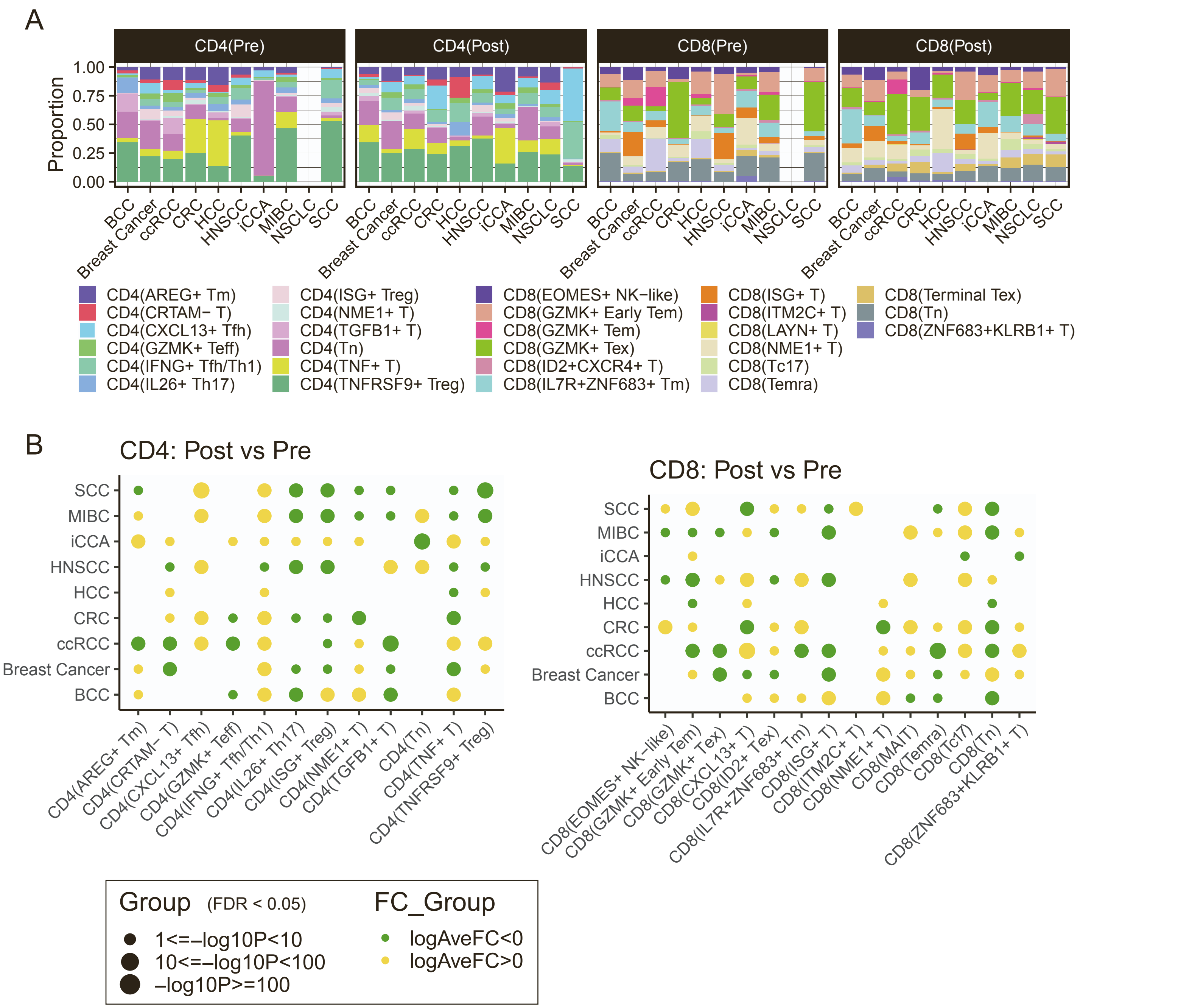

Figure S2. Phenotypic landscapes of treatment-naïve and post-treatment groups. (A) The proportion of cell types across cancer types in treatment-naïve and post-treatment groups. (B) Comparison of the proprtions between the treatment-naïve and post-treatment groups in both CD4/CD8 T cell. Two-proportions z-test was used to compare between the groups. Only comparisons with FDR < 0.05 were shown. Related to Figure 2.

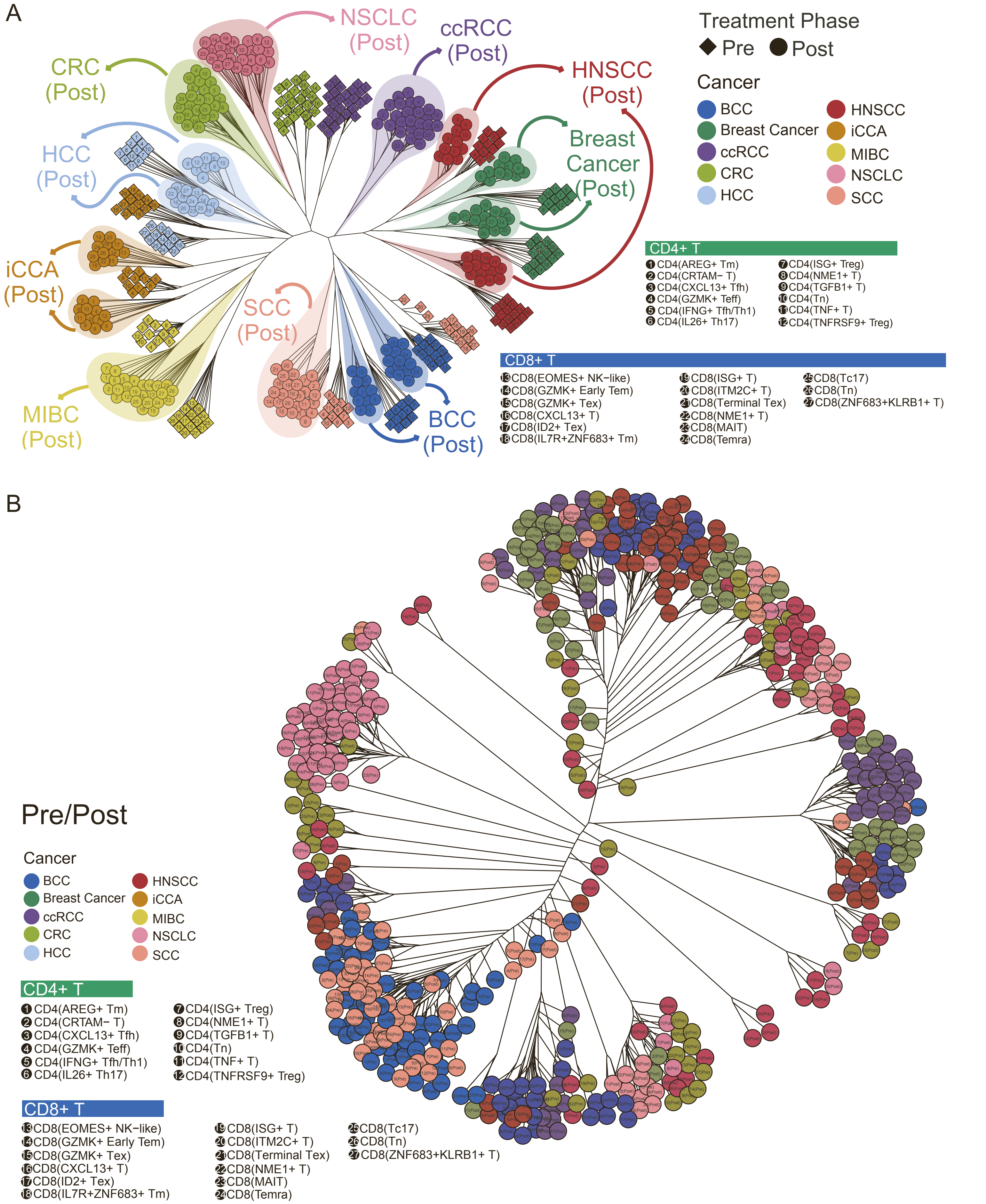

Figure S3. Clustering of TFs from the treatment-naïve and post-treatment groups across cancer types. Clustering of TFs based on their (A) transcriptional activities as well as (B) gene expressions across cancer types in the Pre and Post groups. Related to Figure 2.

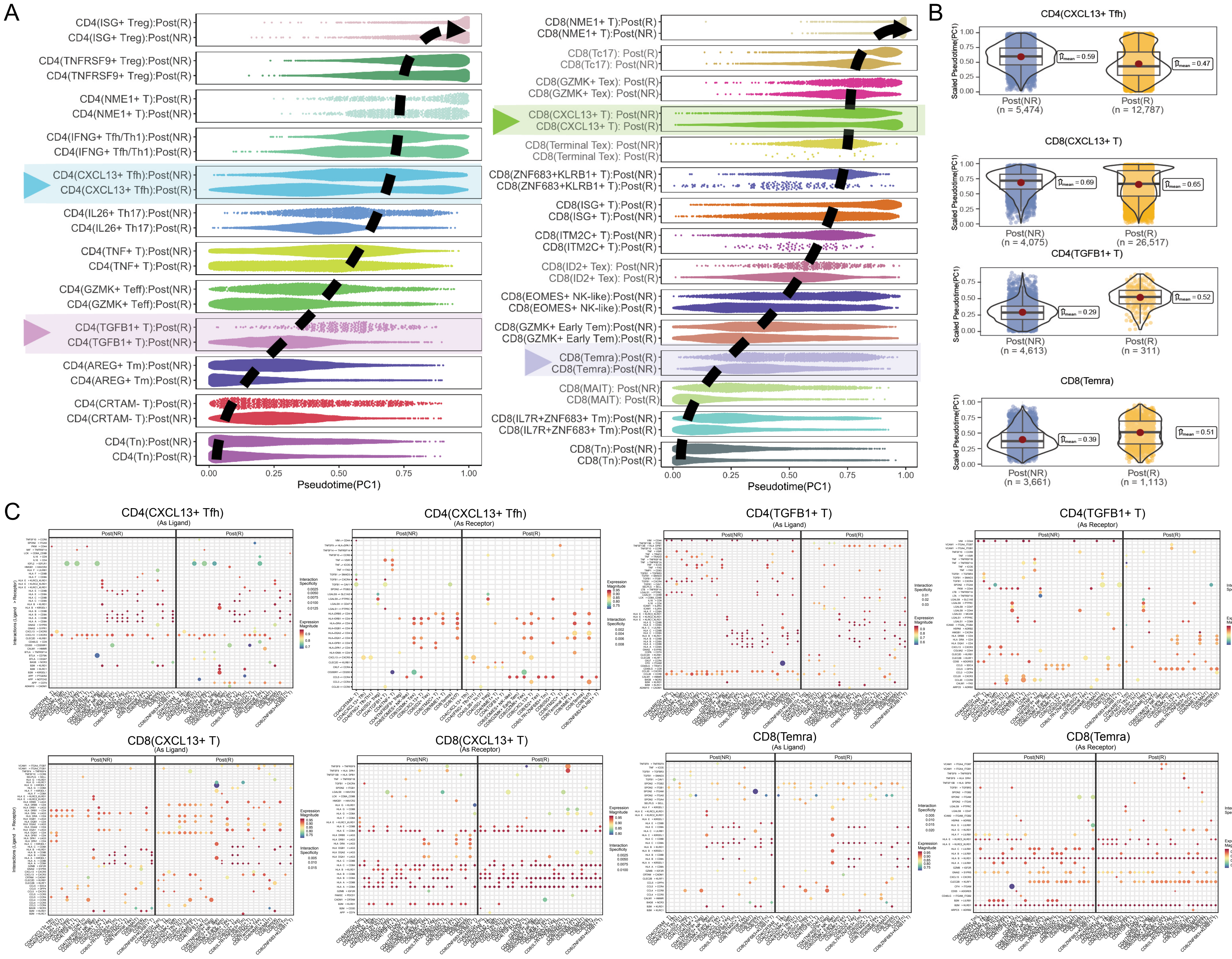

Figure S4. Pseudotime and cell to cell interaction. (A) Pseudotime analysis based on PC1 across all CD4 and CD8 T cells. (B) Box plots showing the result of t-test comparing the pseudotime between the response groups. (C) Top ligand-receptor binding interactions in the IRATs. Size of the dots represented interaction specificity and color gradient indicated the expression of the interaction pairs. Related to Figure 3.

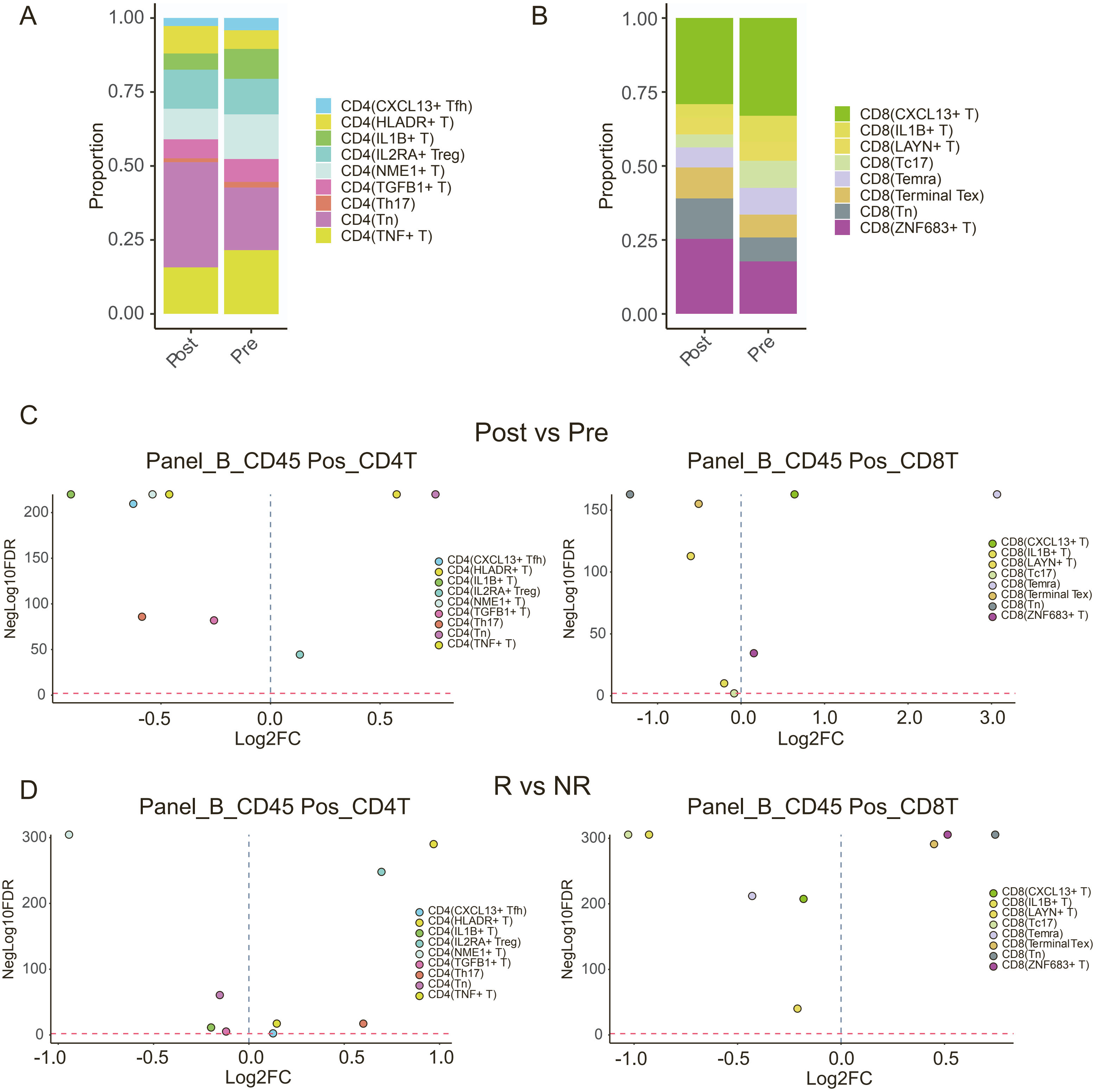

Figure S5. CyTOF proportion analysis result . (A) Proportion of CD4 cell types and (B) CD8 cell types in the treatment-naïve and post-treatment samples. (C) Result of two-samples proportion Z-test between the treatment groups and (D) response groups. Related to Figure 5.
