# Supplementary material for: Deciphering immune predictors of immunotherapy response: A multiomics approach at the pan-cancer level
Source: Cell Rep Med. 2025 Mar 6;6(4):101992. doi: 10.1016/j.xcrm.2025.101992 (PMC12047473; doi:10.1016/j.xcrm.2025.101992)
Supplement: Document S2. Article plus supplemental information [file mmc4.pdf]

Cell Reports
Medicine

Article

Deciphering immune predictors of immunotherapy response: A multiomics approach at the pan-cancer level

Graphical abstract

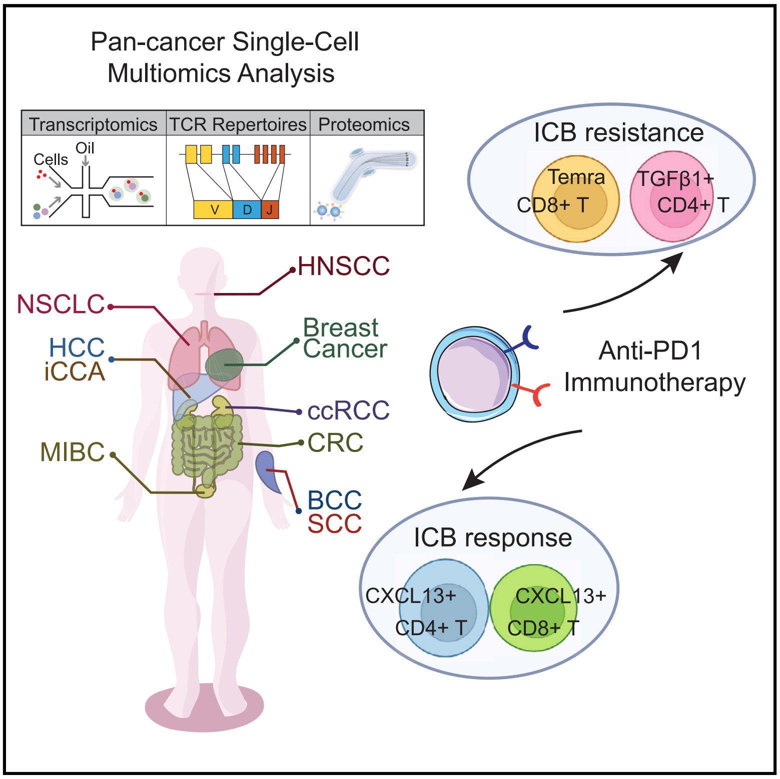

Highlights

- We identify four T cell subsets that influence ICB response across ten cancer types
- We reveal that TGF-β1+ $CD4^+$ and Temra $CD8^+$ T cells correlate with ICB resistance
- We find that CXCL13+ $CD4^+$ and $CD8^+$ T cells correlate with ICB response
- Multiomics approach identifies biomarkers predicting ICB therapy outcomes

Authors

Xuexin Li, Lu Pan, Weiyuan Li, ...,
Xiaolu Zhang, Ren Sheng, Zhenning Wang

Correspondence

xuexin.li@ki.se (X.L.),
xiaolu.zhang@sdu.edu.cn (X.Z.),
shengren@mail.neu.edu.cn (R.S.),
znwang@cmu.edu.cn (Z.W.)

In brief

This study employs single-cell multiomics to reveal T cell dynamics shaping immune checkpoint blockade (ICB) response across cancers. Four T cell subsets associated with ICB responsiveness or resistance are identified, providing immune markers to inform personalized therapies and enhance understanding of ICB mechanisms in cancer treatment.

Li et al., 2025, Cell Reports Medicine 6, 101992
April 15, 2025 © 2025 The Author(s). Published by Elsevier Inc.
https://doi.org/10.1016/j.xcrm.2025.101992

CellPress

Cell Reports Medicine

CellPress
OPEN ACCESS

Article

Deciphering immune predictors of immunotherapy response: A multiomics approach at the pan-cancer level

Xuexin Li,[1,2,3,4,22,23,*] Lu Pan,[5,22] Weiyuan Li,[6,7,22] Bingyang Liu,[8,22] Chunjie Xiao,[6] Valerie Chew,[9] Xuan Zhang,[10] Wang Long,[11] Florent Ginhoux,[12,13,14] Joseph Loscalzo,[15] Marcus Buggert,[16] Xiaolu Zhang,[17,18,*] Ren Sheng,[19,20,*] and Zhenning Wang[2,3,21,*]

[1]Department of General Surgery, The Fourth Affiliated Hospital, China Medical University, Shenyang, Liaoning 110032, China
[2]Key Laboratory of Precision Diagnosis and Treatment of Gastrointestinal Tumors, Ministry of Education, China Medical University, Shenyang, Liaoning 110122, China
[3]Institute of Health Sciences, China Medical University, Shenyang, Liaoning 110122, China
[4]Department of Physiology and Pharmacology, Karolinska Institutet, 171 65 Solna, Sweden
[5]Institute of Environmental Medicine, Karolinska Institutet, 171 65 Solna, Sweden
[6]School of Medicine, Yunnan University, Kunming, Yunnan 650091, China
[7]Department of Reproductive Medicine, The First People's Hospital of Yunnan Province, Kunming, Yunnan 650021, China
[8]Department of Endocrinology, Shengjing Hospital of China Medical University, Shenyang, Liaoning 110004, China
[9]Translational Immunology Institute (TII), SingHealth-Duke NUS Academic Medical Centre, Singapore 169856, Singapore
[10]Department of Colorectal Surgery, Yunnan Cancer Hospital, The Third Affiliated Hospital of Kunming Medical University, Kunming, Yunnan 650032, China
[11]Department of Pathology, Nihon University, Tokyo 102-0074, Japan
[12]Singapore Immunology Network (SIgN), Agency for Science, Technology and Research (A*STAR), Singapore 138648, Singapore
[13]Institut Gustave Roussy, INSERM U1015, Bâtiment de Médecine Moléculaire 114 rue Edouard Vaillant, 94800 Villejuif, France
[14]Shanghai Institute of Immunology, Department of Immunology and Microbiology, Shanghai Jiao Tong University School of Medicine, Shanghai 200025, China
[15]Department of Medicine, Brigham and Women's Hospital, Harvard Medical School, Boston, MA 02115, USA
[16]Center for Infectious Medicine, Department of Medicine Huddinge, Karolinska Institutet, 141 52 Huddinge, Sweden
[17]Department of Physiology and Pathophysiology, School of Basic Medical Sciences, Cheeloo College of Medicine, Shandong University, Jinan, Shandong 250012, China
[18]Shenzhen Research Institute of Shandong University, Shenzhen, Guangdong 518057, China
[19]College of Life and Health Sciences, Northeastern University, Shenyang, Liaoning 110819, China
[20]School of Basic Medical Sciences, Guangzhou Medical University, Guangzhou, Guangdong 510000, China
[21]The First Affiliated Hospital of China Medical University, Shenyang, Liaoning 110001, China
[22]These authors contributed equally
[23]Lead contact
*Correspondence: xuexin.li@ki.se (X.L.), xiaolu.zhang@sdu.edu.cn (X.Z.), shengren@mail.neu.edu.cn (R.S.), znwang@cmu.edu.cn (Z.W.)
https://doi.org/10.1016/j.xcrm.2025.101992

SUMMARY

Immune checkpoint blockade (ICB) therapy has transformed cancer treatment, yet many patients fail to respond. Employing single-cell multiomics, we unveil T cell dynamics influencing ICB response across 480 pan-cancer and 27 normal tissue samples. We identify four immunotherapy response-associated T cells (IRATs) linked to responsiveness or resistance and analyze their pseudotemporal patterns, regulatory mechanisms, and T cell receptor clonal expansion profiles specific to each response. Notably, transforming growth factor β1 (TGF-β1)+ CD4$^+$ and Temra CD8$^+$ T cells negatively correlate with therapy response, in stark contrast to the positive response associated with CXCL13+ CD4$^+$ and CD8$^+$ T cells. Validation with a cohort of 23 colorectal cancer (CRC) samples confirms the significant impact of TGF-β1+ CD4$^+$ and CXCL13+ CD4$^+$ and CD8$^+$ T cells on ICB efficacy. Our study highlights the effectiveness of single-cell multiomics in pinpointing immune markers predictive of immunotherapy outcomes, providing an important resource for crafting targeted immunotherapies for successful ICB treatment across cancers.

INTRODUCTION

The tumor microenvironment (TME) is a complex and dynamic landscape that varies across cancer types.[1] Within this environment, tumor-infiltrating T cells emerge as a critical feature, exhibiting the capacity to both suppress and promote tumor growth.[1–3] Immunotherapy, and in particular immune checkpoint blockade (ICB),[4] is able to enhance cytotoxicity of T cells by

© 2025 The Author(s). Published by Elsevier Inc.
This is an open access article under the CC BY license (http://creativecommons.org/licenses/by/4.0/).

inhibiting checkpoint molecules on their surface,[5] thereby reinvigorating cellular immunity within the TME to curtail cancer growth.[6,7] However, the efficacy of ICB therapies is not uniform across all patients, largely due to several complicating factors such as tumor heterogeneity,[8,9] T cell exhaustion,[9–11] inadequate T cell infiltration,[7] the presence of immune-suppressive elements within the TME,[12] and differences in tumor mutational burden (TMB).[13–15] As such, there is a need to delineate whether common immune signatures exist between cancer types that could facilitate the prediction of ICB efficacy across tumor types.

Recent advancements in integrative single-cell technologies enable detailed analysis and the discovery of T cell subsets in the TME across various cancer types.[16–18] These technologies have facilitated the discovery and subtyping of tumor-infiltrating T cells, particularly those exhibiting different states of exhaustion, and have enabled a more nuanced understanding of their roles in either antagonizing or supporting tumor growth. Importantly, these studies have also begun to shed light on the clinical relevance of these T cells, including their impact on the efficacy of ICB therapies.[19–21] Among the various tumor-infiltrating T cell subsets, CXCL13+ T cells have emerged as a notable subset co-expressing multiple inhibitory receptors, such as programmed cell death protein 1 (PD-1), T cell immunoreceptor with immunoglobulin and ITIM domain (TIGIT), lymphocyte activation gene 3 (LAG3), and cytotoxic T-lymphocyte associated protein 4 (CTLA-4) on CD8$^+$ T cells.[16,22,23] Furthermore, the presence of CXCL13+ T cells has been associated with enhanced anti-PD-1 responses in various cancers such as non-small cell lung cancer (NSCLC),[22,24] high-grade serous ovarian cancer,[25] and others,[23,26,27] where their high infiltration correlates with improved prognoses in certain contexts.[28] Conversely, in conditions like clear cell renal cell carcinoma (ccRCC), a high presence of CXCL13+ CD8$^+$ T cells has been linked to poorer survival outcomes,[29] illustrating the context-dependent nature of these immune responses. This highlights a complex and critical area of research that requires further exploration to understand the differential roles and underlying mechanisms of CD4$^+$ and CD8$^+$ CXCL13+ T cells, especially those with varying levels of exhaustion and their interactions with other tumor-infiltrating T cells in response to ICB treatments.

On another front, transforming growth factor β (TGF-β) introduces another layer of complexity in the TME.[30,31] TGF-β is a cytokine that plays a crucial role in immune evasion, often diminishing the effectiveness of ICB responses by inhibiting T helper (Th) 1 differentiation and directly suppressing the cytotoxic functions of CD8$^+$ T cells.[32] This aspect underscores the need for deeper investigation into the interactions between TGF-β-producing tumor-infiltrating T cells and other T cell subsets, particularly those sensitive to ICB, to uncover novel therapeutic targets.

To date, single-cell research into tumor-infiltrating T cells has largely focused on dissecting tissue-specific cancer types.[28,33,34] As such, there remains an urgent need to discover common and unique ICB-responsive tumor-infiltrating T cell subsets across different cancers, understand their immunological landscape, and elucidate their specific interactions and evolutionary trajectories within the context of ICB response. This comprehensive approach is essential for advancing our understanding of the mechanisms driving ICB efficacy and for developing more effective immunotherapeutic strategies. Specifically, in the context of colorectal cancer (CRC), while significant strides have been made in identifying general tumor-infiltrating T cell populations, the integration of multi-layered omics analyses in post-ICB-treated patients represents a largely untapped area of research.[28,35] Such integrative efforts are crucial for gaining a deeper understanding of the immunological dynamics within CRC and could pave the way for the development of more precise and efficacious treatment modalities, ultimately enhancing patient outcomes in the face of this challenging disease.

To meet these challenges, we developed a comprehensive single-cell multiomics atlas covering ten cancer types and three omics layers, focusing on patients undergoing immunotherapy and changes to tumor-infiltrating T cells based on their therapy responses, which followed the response evaluation criteria in solid tumours v.1.1 (RECIST v.1.1) criteria.[36] This allowed us to pinpoint both known and novel tumor-infiltrating T cell subsets that are specific to certain cancers, their response to therapy, or lack thereof. We examined the molecular regulation and cellular interactions among these T cells, identifying immune profiles and T cell receptor (TCR) repertoires unique to specific cancer types or their response to treatment. Additionally, we investigated the survival impact of these T cells post ICB therapy and evaluated their potential as biomarkers for predicting treatment outcomes. This resource extends beyond previous work by uncovering new tumor-infiltrating T cell populations also in ICB-treated patients with CRC through the usage of mass cytometry, identifying the key cell types in modulating ICB response.

RESULTS

Characteristics of pan-cancer single-cell dataset across cohorts

We first compiled an extensive multiomics dataset of single T cells across ten cancer types, including basal cell carcinoma (BCC), squamous cell carcinoma (SCC), breast cancer, ccRCC, CRC, hepatocellular carcinoma (HCC), head and neck SCC (HNSCC), intrahepatic cholangiocarcinoma (iCCA), muscle-invasive bladder cancer (MIBC), and NSCLC (Figure 1). We organized these datasets into three "cohorts": the pan-cancer transcriptomics atlas (discovery cohort 1) comprising single-cell RNA sequencing (scRNA-seq) data; the pan-cancer immune atlas (discovery cohort 2) constituting single-cell immune profiling (scImmune profiling) data containing cancer samples with scImmune profiling data from discovery cohort 1 and in addition, normal samples corresponding to the primary sites of the cancer types included; and a validation cohort for CRC with single-cell proteomics mass cytometry—cytometry by time of flight (CyTOF) data. After post-quality control and batch corrections, discovery cohort 1 consisted of 795,645 cells from 244 samples/126 patients across ten cancer types.[37] This cohort was divided into treatment-naive (pre, 83 samples/71 patients) and post-ICB treatment (post, 161 samples/108 patients) samples and further classified into clinically responsive (ICB-R, 57 samples/33 patients) and non-responsive (ICB-NR, 50 samples/27 patients) groups based on the RECIST v.1.1 criteria

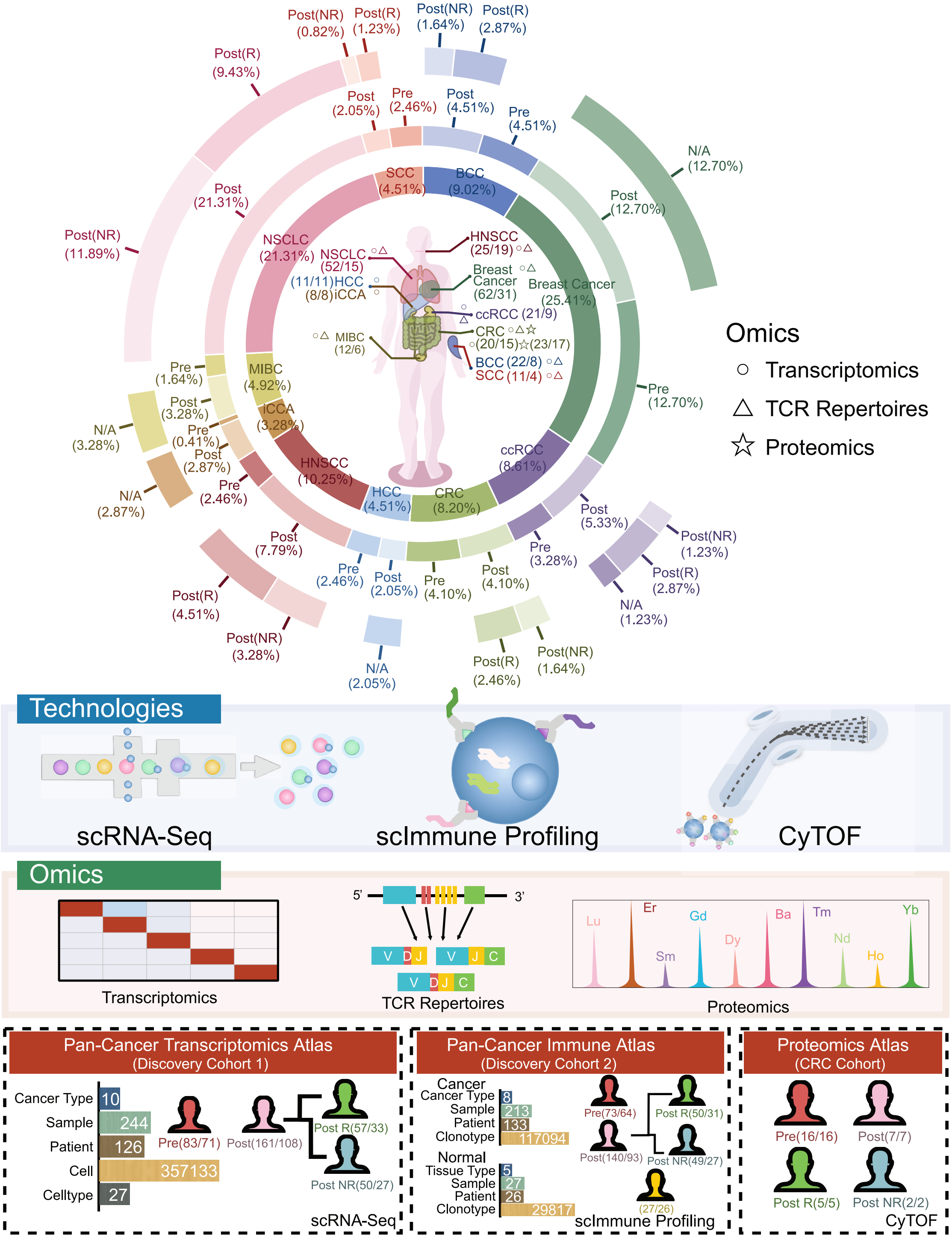

Figure 1. Demographic overview of the pan-cancer T cell atlas

In the donut diagram, numbers in the brackets represent sample number/patient number. The atlas comprised three major cohorts, namely, the pan-cancer transcriptomics cohort, the pan-cancer immune cohort, and the CRC cohort.

(Figures 1 and 2A).[36] Discovery cohort 2 consisted of scImmune profiling data from 213 samples/133 patients across eight cancer types and 27 samples/26 individuals from healthy tissues (Figure 1; Tables S1 and S2). The CRC validation cohort, smaller in size (23 samples/23 patients), involves a more in-depth single-cell proteomics analysis (Figure 1; Tables S1 and S2).

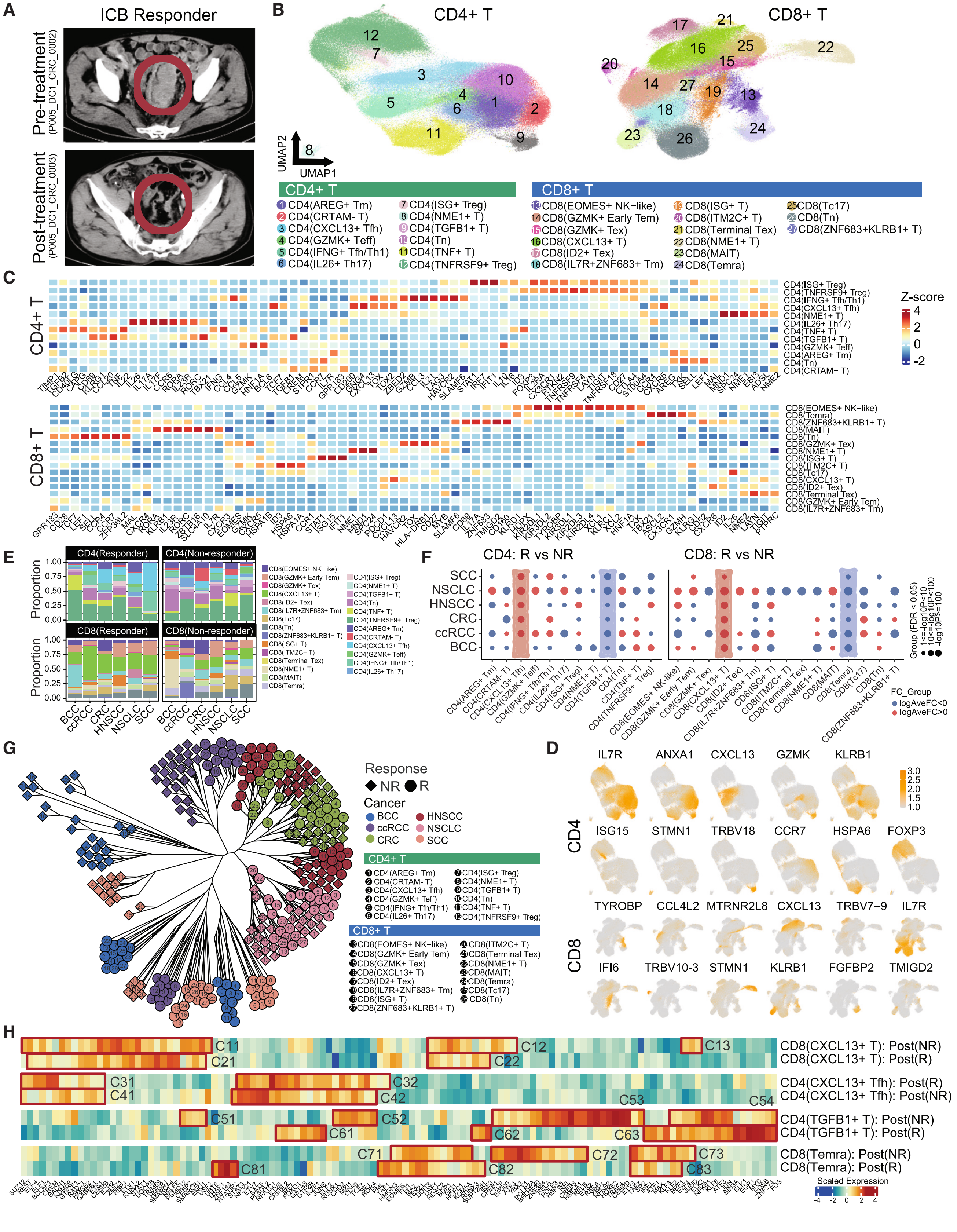

(legend on next page)

CellPress
OPEN ACCESS

In this comprehensive investigation, we elucidated the complex landscape of tumor-infiltrating T cell dynamics across the spectrum of cancers, revealing subsets that exhibit distinct responses to ICB therapy. Leveraging the extensive dataset of 480 pan-cancer and 27 normal tissue samples from the three cohorts of data and with the aid of cutting-edge single-cell omics technologies, we identified and validated four key T cell subsets marked by their unique responsiveness or resistance to ICB therapy. Our analysis illuminated the pseudotemporal evolution, regulatory mechanisms, and TCR clonal expansion patterns specific to each of these subsets and provides a foundational understanding of the dynamic behavior of tumor-infiltrating T cells in response to immunotherapy.

Pan-cancer analysis identified four immunotherapy response-associated T cells

In our investigation, we discerned a diverse array of T cell subtypes from the ten cancer types, comprising 12 distinct $CD4^+$ and 15 $CD8^+$ T cell subsets (Figure 2B) based on signature genes (Figures 2C and 2D). Among these, $CD8^+$ T cell subsets, each representing different phases of $CD8^+$ T cell exhaustion within the TME, were found (Figure S1),[16] corroborating with earlier studies.[18] Our analysis next began with the meticulous identification of T cell subsets that changed according to treatment, followed by the discovery of T cells that exhibited specific responses to ICB treatment across a diverse array of cancers using discovery cohort 1.

Analyzing the variance in T cell subtype distribution before and after immunotherapy revealed notable trends. Specifically, an increase in $CD4^+$ TNF receptor superfamily Member 9 (TNFRF9+) regulatory T cells was observed in cancers such as iCCA, ccRCC, and BCC following ICB therapy. Additionally, a pan-cancer surge in $CD4^+$ interferon gamma (IFNG+) T follicular helper (Tfh)/Th1 cells, $CD8^+$ Tc17 cells, and mucosal-associated invariant T cells was noted post ICB treatment (Figures S2A and S2B).

The dataset was further stratified based on ICB responsiveness, revealing response-specific gene sets, as well as significant changes in the T cell compositions within the TME attributed to ICB response (Figures 2E and 2F). Although the impact of ICB therapy responsiveness induced a wide array of changes across nearly all examined T cell types, four immunotherapy response-associated T cells (IRATs) demonstrated pan-cancer consistency in their compositional shifts. $CD4^+$ CXCL13+ Tfh cells and $CD8^+$ CXCL13+ T cells predominantly expanded among ICB-Rs, which we termed IRATs as tumor-responsive T cells (TRTs). In contrast, an increase in $CD4^+$ TGF-β1+ T cells and $CD8^+$ Temra cells was significantly noted in ICB-NRs (Figures 2E and 2F), which we named tumor-non-responsive T cells (TNRTs). This observation aligns with previous research indicating that the expansion of precursor-exhausted $CD8^+$ (CXCL13+ T) and CXCL13+ Th1-like cells is associated with responsive tumors and correlates with favorable ICB outcomes.[22,24,25] Conversely, a lower abundance of $CD8^+$ Temra cells has been linked to improved responses to immunotherapy.[38] Moreover, the immunosuppressive role of TGF-β1, which was known to facilitate tumor progression and evasion from immune surveillance, underscores the significance of these compositional changes. The consistent alteration in these IRATs across different cancers highlights their potential as biomarkers for predicting ICB treatment response. Understanding the mechanisms behind their expansion or reduction in the context of ICB therapy may unlock new avenues for enhancing immunotherapeutic efficacy and personalizing cancer treatment strategies.

Distinct regulatory patterns of tumor-infiltrating T cells through gene regulatory network analysis

Our investigation into the gene regulatory networks governing tumor-infiltrating T cell regulations unveiled distinct regulatory mechanisms at play across cancer types. Utilizing single-cell regulatory network inference and clustering analysis[39] to delineate the transcription factor (TF) activities within each T cell subtype, we embarked on a comprehensive exploration of their regulatory landscapes.

Hierarchical clustering based on cell-type-specific TF activities for post-ICB samples demonstrated that, regardless of cell subtypes, cell types are closely clustered based on their cancer types and response groups, with a clear inter-group clustering pattern of $CD4^+$ and $CD8^+$ T cell subsets, and cancers of analogous tissue origins were also in close proximity—for instance, skin-derived BCC and SCC, as well as liver cancers HCC and iCCA (Figure 2G). Similar findings were also observed for assessing the samples in terms of their treatment groups (Figure S3A). This illustrates that regulatory elements in different T cell subsets dictate similar regulatory affinities within cancer types and tissue origins, while treatment interventions, as well as responsiveness to ICB, could indeed cause regulatory responses in a cancer- and tissue-origin-specific way (Figures 2G and S3A). Furthermore, extending our analysis to include the gene expression profiles of the TFs brought to light a greater diversity such that clustering effects within cancer types and treatment groups have diminished (Figure S3B), underscoring the nuanced regulation within the TME and affirming the profound influence of ICB therapy on T cell regulatory dynamics.

Figure 2. Pan-cancer T cell landscape in the transcriptomics cohort

(A) Tomographic images of a CRC responder taken before and 6 weeks after ICB treatment.

(B) Uniform manifold approximation and projection (UMAP) representations of $CD4^+$ and $CD8^+$ T cells.

(C) The proportion of cell types across cancer types in ICB-R and ICD-NR groups. Cancer types included BCC, SCC, ccRCC, CRC, HNSCC, and NSCLC.

(D) Summary statistics, including FDR and fold change (FC) of $CD4^+$ and $CD8^+$ T cells (in log scale), for the proportion test between the ICB-R and ICB-NR groups in both $CD4^+$/$CD8^+$ T cells. Two-proportion z-test was used to compare between the groups. Only comparisons with FDR < 0.05 were shown.

(E) Heatmap showing standardized expression of the signature markers in $CD4^+$ and $CD8^+$ T cells.

(F) UMAP representations of the expression of signature markers of $CD4^+$ and $CD8^+$ T cells across all cell types.

(G) Clustering of TFs based on their transcriptional activities across cancer types in ICB-R and ICB-NR groups.

(H) TF signatures of the IRATs in each response group.

Elucidating the regulatory patterns of the IRATs correlating ICB response

Our analysis extended into the TF regulation to discern gene expression patterns of the IRATs distinctively associated with response to ICB therapy. The focused comparison of TF expressions between ICB-Rs and ICB-NRs unveiled TFs intimately linked to the ICB treatment responsiveness (Figure 2H).

Notably, TF module C53 was markedly upregulated in CD4$^+$ TGF-β1+ T cells among ICB-NRs, encompassing several nuclear factor κB (NF-κB) pathway genes (*REL*, *RELA*, *RELB*, *NFKB2*, and *CEBPD*), signifying an active NF-κB signaling pathway in these cells. In a parallel finding, CD8$^+$ Temra T cells from ICB-NRs demonstrated upregulation in the NF-κB family genes (*REL*, *RELA*, and *RELB*) within TF module C72 (Figure 2H). This pattern indicates that NF-κB-mediated signaling is a common characteristic of these T cell subsets in ICB-NRs. Furthermore, genes associated with cell survival and activity (*BHLHE40*, *ATF4*, *EP300*, and *CTCF*) were also elevated in C72, pointing to an enhanced survival mechanism in CD8$^+$ Temra cells within the ICB-NRs. Conversely, CD8$^+$ Temra cells in ICB-Rs exhibited upregulation in module C81, containing *THAP1* and *ZNF143* (Figure 2H), suggesting a different set of regulatory mechanisms that might influence the proliferation of this subset within the TME. The elucidation of these response-specific TF modules sheds light on the complex regulatory landscape governing the behavior of IRATs in the context of ICB response.

IRATs undergo significant pseudotemporal changes after immunotherapy

We next explored the temporal dynamics of the IRATs by modeling their developmental trajectories using Monocle2[40] and systematically evaluated the pathways specific to responsiveness in each trajectory (Figures 3D and 3E). Both CD4$^+$ TGF-β1+ T cells and CD8$^+$ Temra cells exhibited divergent differentiation in ICB-NRs, characterized by unique branch pathways (Figures 3D and 3E).

Notably, in ICB-NR states, there was an observed increase in pathways that were decreased in ICB-Rs, such as those related to apoptosis and hypoxia. CD4$^+$ CXCL13+ Tfh cells and CD8$^+$ CXCL13+ T cells demonstrated a more complex differentiation state in ICB-Rs, developing into multiple functional pathways, in stark contrast to the simpler differentiation observed in ICB-NRs. Despite this, there were some commonalities in the pathways between the two response groups, such as the involvement of IFN-alpha/gamma responses and reduced tumor necrosis factor alpha (TNF-α) signaling via NF-κB in specific branches.

Our exploration delved into the pseudotemporal dynamics of the IRATs, seeking to understand how these dynamics correlate with responses to ICB. Utilizing pseudotemporal analysis, we traced the differentiation pathways of these IRATs and compare their trajectories between the ICB-Rs and ICB-NRs (Figures 3A–3D, S4A, and S4B). The analysis revealed that TRTs, which are IRATs associated with ICB-Rs including CD4$^+$ CXCL13+ Tfh cells and CD8$^+$ CXCL13+ T cells, followed a more rapid pseudotemporal differentiation trajectory compared to TNRTs (Figure 3A). TNRTs CD4$^+$ TGF-β1+ T cells and CD8$^+$ Temra cells demonstrated a notably slower development rate in the ICB-Rs (Figures 3A and S4B), suggesting a correlation between delayed differentiation and less favorable ICB response outcomes, underscoring a potential mechanistic link between accelerated TRT evolution and positive ICB therapy response.

Employing Monocle2 for modeling developmental trajectories, we systematically assessed pathways specific to responsiveness within each trajectory (Figures 3B–3D). Our findings highlighted divergent differentiation paths for IRATs in ICB-Rs and ICB-NRs, marked by distinctive branch pathways (Figures 3B and 3C). Notably, an inverse regulatory pattern was observed between ICB-Rs and ICB-NRs, such that the upregulated pathways in the pseudotime states of ICB-NRs (Figures 3B and 3C) were downregulated in ICB-Rs, including those related to apoptosis and hypoxia. In TRTs, CD4$^+$ CXCL13+ Tfh and CD8$^+$ CXCL13+ T cells exhibited more complex differentiation in ICB-Rs, branching into multiple functional pathways. This complexity might be suggesting that these TRTs possessed a broader and more nuanced role in mediating successful immunotherapy responses. Despite these divergent patterns, some shared pathways emerged between ICB-Rs and ICB-NRs, indicating common regulatory mechanisms at play, such as IFN-alpha/gamma responses and diminished TNF-α signaling via NF-κB in specific subsets (Figures 3B–3D).

Deciphering cell-cell interactions of IRATs

Additionally, a significant presence of TNF superfamily ligand-receptor interactions was observed in CD4$^+$ TGF-β1+ T cells with other tumor-infiltrating T cells in ICB-NRs (Figure S4C). These results underscore the complex interplay of immune responses in the TME and may provide insights into potential therapeutic targets.

In our comprehensive analysis, we delved into the intricate cell-cell interactions among the IRATs (Figures 3E and S4C). Utilizing CellPhoneDB,[41] we mapped the complex web of interactions that delineate the roles of these T cells in ICB responses (Figure 3E). Globally, IRATs generally exhibited an enhanced level of interactions with other T cells in the TME of the ICB-Rs (Figure 3E), pointing toward a synergistic immune activation conducive to positive therapy outcomes. Interestingly, CD8$^+$ Interferon-stimulated genes (ISGs+) T cells, known for their potent anti-tumor properties,[16,42] emerged as primary interactors with CD4$^+$ CXCL13+ Tfh, CD8$^+$ CXCL13+ T, and CD8$^+$ Temra cells in the ICB-Rs. This interaction pattern underscores the critical role of CD8$^+$ ISG+ T cells in orchestrating effective anti-tumor responses with these IRATs. On the other hand, CD4$^+$ TGF-β1+ T cells were more inclined toward increased interactions within the ICB-NRs rather than in the ICB-Rs. Notably, these cells frequently interacted among themselves and with CD4$^+$ cytotoxic and regulatory T Cell molecule (CRTAM−) T cells (Figure 3E), suggesting a network that may underpin resistance mechanisms.

Inspecting across the top ligand-receptor interactions of the CD4$^+$ TGF-β1+ T cells, prevalent CCL20/CCR6 and CCL20/CXCR3 interactions were observed with almost all other T cells in the ICB-NRs (Figure S4C), highlighting a potential mechanism for promoting a Th17 response and contributing to therapeutic resistance.[43] Furthermore, a significant presence of TNF superfamily ligand-receptor interactions was also observed in CD4$^+$ TGF-β1+ T cells with other T cells within the ICB-NRs (Figure S4C), indicating another layer of complexity in the immune evasion strategies employed by resistant tumors.

CellPress
OPEN ACCESS

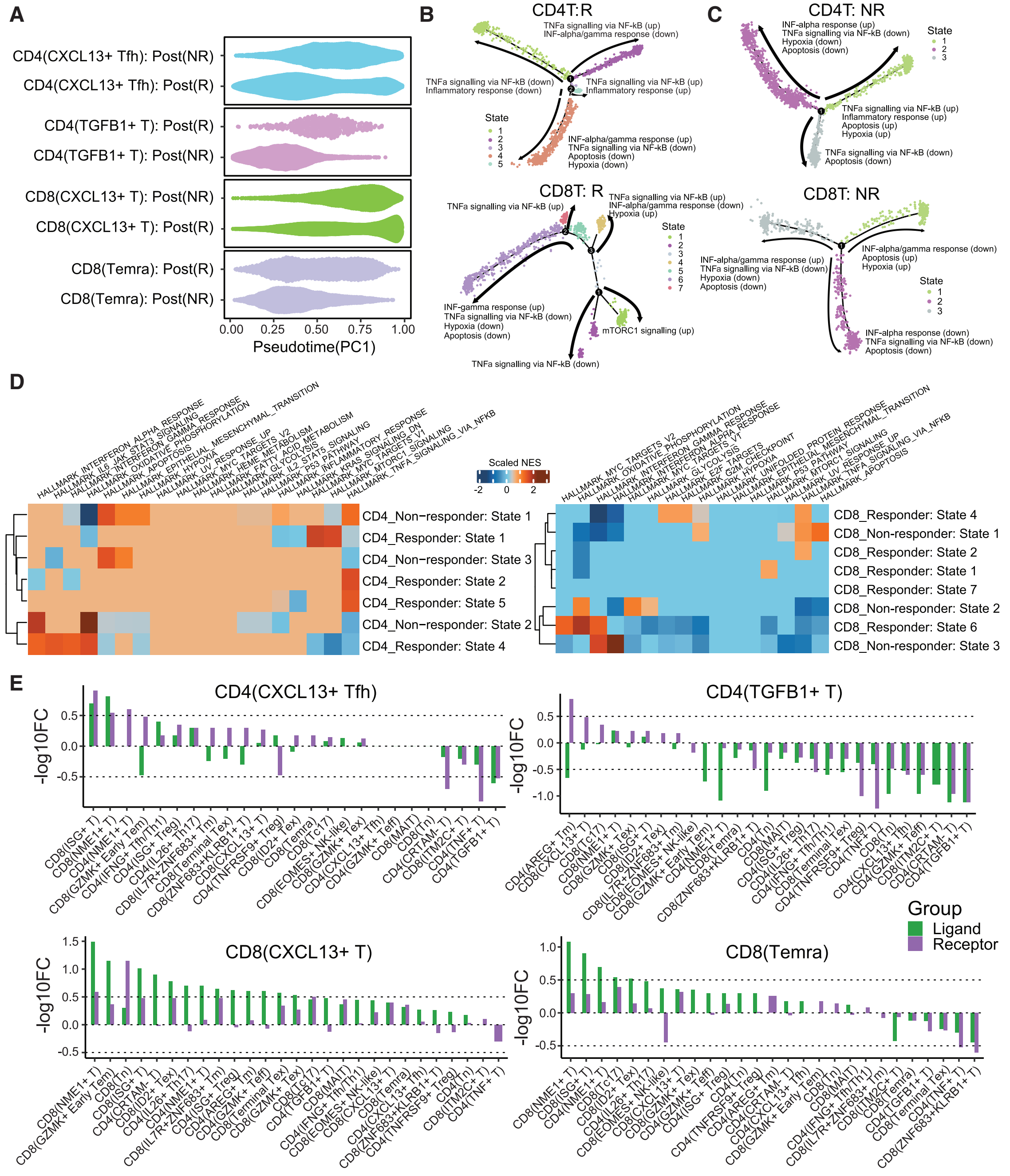

Figure 3. T cell landscapes of the responders and non-responders in the transcriptomics cohort

(A) Pseudotime of the IRATs in the response groups.

(B and C) Enriched pathways in each of the pseudotime branches for the IRATs.

(D) Normalized enrichment scores (NESs) of the enriched hallmark pathways in each pseudotime branch of the IRATs in (B) and (C).

(E) Cell-cell interaction frequencies of the IRATs with all CD4+/CD8+ T cells. y axis displays the $\log_{10}$FC (represented in $-\log_{10}$FC scale) in the number of ligands or receptors facilitating cell-cell interactions between the specified cell types (noted in the plot subtitles) and the cell types shown on the x axis. The numerator of the fold change represents cells from responders, while the denominator represents cells from non-responders.

The pan-cancer immune atlas—Discovery cohort 2

Focusing on TCR repertoires and clonal homeostasis, we uncovered consistent patterns of clonal expansion across different cancers and response groups. Leveraging these insights, we further developed a multiomics index based on the scRNA-seq profile from discovery cohort 1 and immune profiles from discovery cohort 2 of the IRATs, achieving high accuracy, sensitivity, specificity, and precision in predicting ICB responses.

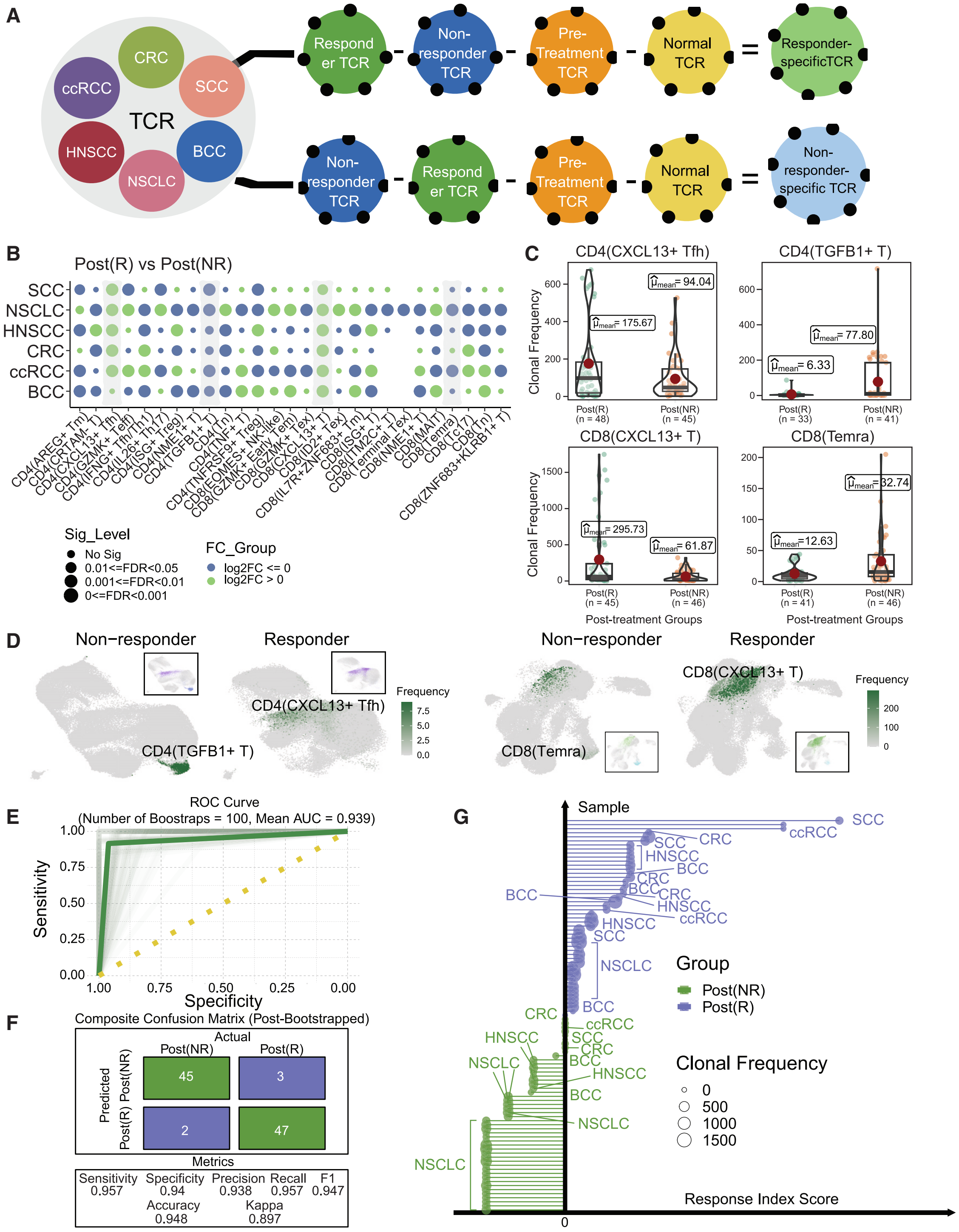

Figure 4. Pan-cancer immune cohort demonstrated an expansion in the IRATs

(A) Workflow for obtaining response-specific TCRs.

(B) Fold change of TCR clonal frequency between the R (numerator) and the NR (denominator) groups. Fisher exact test was used to assess the proportional difference between the groups.

(C) Violin plot displaying the comparison between the response groups of the IRATs based on t test comparison.

(legend continued on next page)

CellPress
OPEN ACCESS

TCR repertoire analysis reveals distinct clonal expansion patterns in IRATs

Building on the foundational insights from discovery cohort 1, which highlighted distinct mechanistic patterns of the IRATs, we shifted our focus toward the functional dynamics of these cells, particularly in terms of clonal expansion. This process is pivotal for mounting a precise immune defense, enabling the selective amplification of IRATs equipped with receptors specific to the antigens encountered, thus ensuring a potent and directed immune response. In our detailed exploration, we sought to unravel ICB-responsiveness-specific TCR repertoires across different cancer types, paying special attention to the IRATs and their correlation with ICB responses.

By comparing the TCR repertoires among ICB-Rs, ICB-NRs, treatment-naive, and normal samples (Figure 4A) of discovery cohort 2, which includes cancer samples with scImmune profiling data from discovery cohort 1, along with normal samples corresponding to the primary sites of the included cancer types, we delineated unique TCR sequences exclusive to each group of samples, identifying 39,691 responder-specific and 44,964 non-responder-specific sequences. Linking TCR sequencing data with scRNA-seq insights from discovery cohort 1 allowed us to delve into the clonal expansion nuances within the IRATs. Figures 4B and 4C underscore the remarkable consistency in TCR profiles across a spectrum of cancers for the identified IRATs. We observed that TNRTs CD4$^+$ TGF-β1+ T cells and CD8$^+$ Temra cells predominantly expanded in ICB-NRs, mirroring their increased presence. Conversely, TRTs CD4$^+$ CXCL13+ Tfh cells and CD8$^+$ CXCL13+ T cells were notably more expansive in ICB-Rs, aligning with the compositional shifts detected in discovery cohort 1 (Figure 4B). An intriguing anomaly was noted in CRC responders, where CD8$^+$ Temra cells displayed a marked increase in CRC TCR clonal expansion, diverging from the consistent trends observed in other cancers (Figure 4B).

On the other hand, the variability in expansion patterns across non-IRATs revealed no uniform trends, suggesting a complex landscape of T cell responses to ICB therapy. At the pan-cancer levels, pronounced increase in clonality among CD4$^+$ TGF-β1+ T cells and CD8$^+$ Temra cells in ICB-NRs could be seen based on their TCR clonality (Figures 4C and 4D). In contrast, enhanced clonality was evident for CD4$^+$ CXCL13+ Tfh cells and CD8$^+$ CXCL13+ T cells in ICB-Rs, showing distinctive TCR dynamics at play in the ICB response to cancer.

Leveraging IRATs for predicting ICB response: A multiomics approach

We deployed the transcriptomic profiles (discovery cohort 1) and TCR expansion patterns of the IRATs (discovery cohort 2) from the two discovery cohorts to explore the predictive capacity of IRATs for ICB responsiveness. Our objective was to ascertain if IRATs could reliably forecast ICB responsiveness, positing them as potential prognostic markers.

Utilizing data from discovery cohorts 1 and 2, we constructed a multiomics response index (RI). This involved identifying response- and cell-type-specific pathways from the Molecular Signatures Database,[44] coupled with analyzing the clonal expansion fold change between ICB-Rs and ICB-NRs. We employed a generalized linear model with a ridge penalty for the construction of the prediction model for the RI and segmented our dataset into a 70% training subset and a 30% testing subset. For the training set, the model was further refined through 100 iterations of bootstrapping to enhance the robustness and reliability of the model, which yielded a remarkable average area under the receiver operating characteristic curve of 0.967 (Figures 4E and 4F), underscoring the high predictive accuracy of our model.

Classification was determined through a majority vote across bootstrapping iterations, achieving a final accuracy rate of 0.969, marked by notable sensitivity, specificity, and precision (Figure 4F). This stratification process culminated in the development of a final RI, effectively distinguishing samples based on their likelihood of belonging to either ICB-R or ICB-NR, as showcased in Figure 4G. Notably, the RI demonstrated consistent results across various cancer types, hinting at the feasibility of devising a tailored cancer-specific scoring system for predicting ICB response.

CRC cohort validated the presence of IRATs in post-ICB-treated PBMCs using CyTOF

To validate IRATs found in discovery cohorts 1 and 2, we employed CyTOF to analyze peripheral blood mononuclear cells (PBMCs) from the CRC cohort. This cohort included 23 samples from both treatment-naive patients and those who had received anti-PD-1 therapy, accompanied by clinical response data to facilitate a nuanced analysis of T cell compositions in response to treatment.

Our CyTOF analysis aimed to ascertain whether the patterns of IRATs identified in the TME were mirrored in the translational aspects using the PBMCs from patients with CRC. By examining marker expressions, we delineated nine CD4$^+$ T cell types and eight CD8$^+$ T cell types (Figure 5A), noting differential proportions between responsiveness and treatment groups (Figures 5B and S5). Consistent with the TME observations from discovery cohort 1, TRTs CD4$^+$ CXCL13+ Tfh cells and CD8$^+$ CXCL13+ T cells were found in greater abundance in ICB-Rs compared to ICB-NRs (Figures 5B and S5D). Conversely, TNRTs CD4$^+$ TGF-β1+ T cells and CD8$^+$ Temra cells exhibited significant expansion in the ICB-NRs (Figures 5B and S5D), indicating a parallel in cellular dynamics between peripheral blood and tumor samples.

Further exploring the pseudotemporal patterns, we modeled the developmental trajectories of CD4$^+$ and CD8$^+$ T cell lineages, comparing the average pseudotime of the IRATs between response groups (Figures 5C and 5D). This analysis highlighted some overlapping trends in the CRC CyTOF dataset as compared to what was derived from discovery cohort 1.

(D) Clonal expansion mapped onto the UMAP of CD4$^+$/CD8$^+$ T cells. Color represented the level of clonal frequency. The small UMAP diagrams depicted the location of the IRATs (indicated by their respective cell type colors) among all cells.
(E) Mean receiver operating characteristic (ROC) curve for post-bootstrapping (n = 100) prediction results.
(F) Confusion matrix of the final prediction model.
(G) Final response index across cancer types.

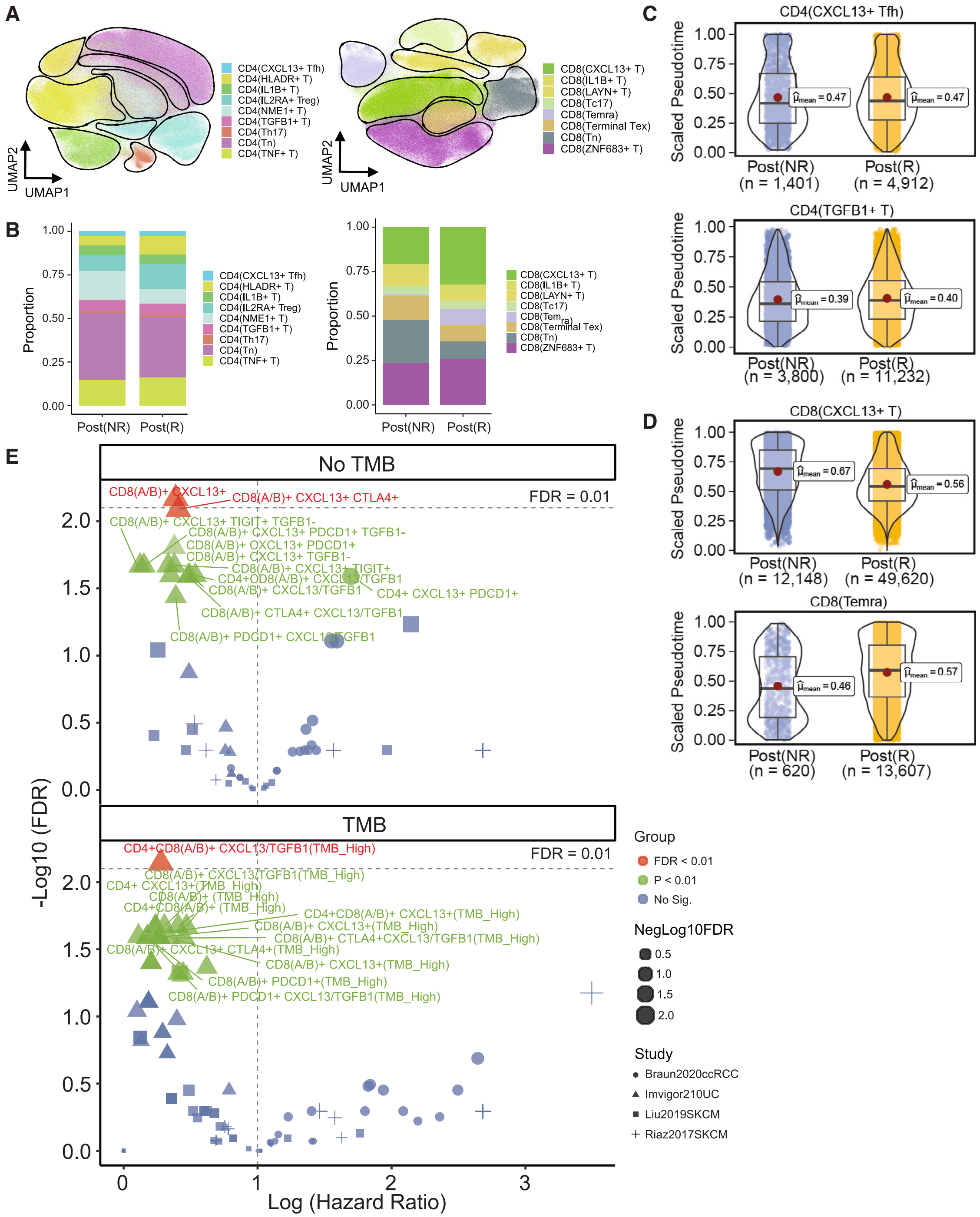

Figure 5. Phenotypic landscape of the validation in CRC cohort and survival analysis on external datasets

(A) UMAP representations of the CD4/CD8 T cells found in the CRC CyTOF dataset.

(B) The abundance of cell types in each response group.

(C and D) Comparison of the abundance between the response groups for the IRATs.

(E) Volcano plots showing Cox hazard ratio (HR) in log scale of the survival analysis based on the expression group of the signature genes in IRATs. Log (HR) > 1 indicates that there is higher risk/worse survival in the cell type expressing these signature genes, while log (HR) < 1 indicates that the cell type presents a protective measure in terms of patient survival.

Specifically, we observed shorter average pseudotimes for $CD8^+$ CXCL13+ T cells among the ICB-Rs and TNRTs $CD4^+$ TGF-β1+ T cells and $CD8^+$ Temra cells showing reduced differentiation rates in ICB-Rs (Figures 5C and 5D). However, no significant pseudotime differences for $CD4^+$ CXCL13+ Tfh cells were observed in the CyTOF data.

These findings in PBMC samples reinforce the significance of IRATs, particularly $CD8^+$ CXCL13+ T cells, $CD4^+$ TGF-β1+ T cells, and $CD8^+$ Temra cells, in reflecting the response of the immune landscape translationally to ICB therapy in the blood compartment. The pseudotime development of $CD4^+$ CXCL13+ Tfh cells in the blood might indicate that the development of $CD4^+$ CXCL13+ Tfh cells might be residential, i.e., occurs locally in the tumor compartment. This cross-validation underscores the robustness of identified IRATs as markers for predicting ICB treatment outcomes, linking tumor dynamics to peripheral immune signatures.

The influence of IRATs on survival outcomes

Our final objective was to investigate how IRATs impact survival outcomes following ICB therapy. While we faced challenges in gathering external CRC immunotherapy data with prognostic information, we successfully obtained comprehensive datasets from urothelial cancer (UC) Imvigor210 cohort with 348 patients with UC[45] and Braun2020 ccRCC dataset with 181 patients,[46] complementing our cohort 1 MIBC and ccRCC findings, as well as two skin cancer datasets, including Liu2019 skin cutaneous melanoma (SKCM) with 121 patients[47] and Riaz2017 SKCM dataset with 50 patients.[21] These datasets provided a robust basis for analyzing the prognostic significance of tumor-infiltrating lymphocytes (TIL) expression patterns. Utilizing survival analysis and leveraging signature genes for each T cell type, we stratified patients into high or low-expression groups based on maximally selected rank statistics.[48] Our survival analysis was also bifurcated to assess the impact of TMB on patient outcomes, creating two distinct cohorts: one accounting for TMB information and the other not.

Survival analysis without considering TMB status

In the cohort where TMB was not considered, patients displaying elevated levels of $CD8^+$ CXCL13+ T cells were observed to have significantly enhanced survival outcomes, particularly notable within the UC cohort. This advantage persisted across variations in $CD8^+$ CXCL13+ T cells with exhaustion marker expression and low expression of TGF-β1, including CTLA4 and programmed cell death protein 1 (PDCD1), indicating that the presence of these $CD8^+$ CXCL13+ T cells was a robust predictor of improved survival independent of T cell exhaustion status. Conversely, an increase in $CD4^+$ TGF-β1+ T cells was invariably linked to diminished survival rates, underscoring their negative prognostic value (Figure 5E). On the other side, in Liu2019, patients with high expression of $CD8^+$ Temra cells faced lower survival rates, whereas those with increased levels of $CD4^+$ CXCL13+ Tfh cells showed improved survival outcomes.

Survival analysis incorporating TMB status

On the other hand, in the cohort considering TMB status, patients characterized by a high ratio of CXCL13 to TGFB1 among $CD4^+$CD8(A/B)+ T cells demonstrated notably higher significance in survival outcomes (marked by $CD4^+$CD8(A/B)+ CXCL13/TGFB1(TMB_High) in Figure 5E), alongside with specific $CD4^+$ or $CD8^+$ T cells marked by high CXCL13 expression, and those with exhaustion markers such as PDCD1 (e.g., CD8(A/B)+PDCD1+CXCL13+(TMB_High)) also showed a significant survival advantage.

Not all IRATs are significantly correlated (Benjamini-Hochberg false discovery rate [FDR] < 0.01) with survival, and their survival correlation varies across these datasets (Figure 5E). These findings underscore significant links between the presence of specific IRATs (with or with considering TMB) with survival following ICB therapy only in specific cancer types and emphasizes the value of considering TMB as a factor in survival predictions.

DISCUSSION

Immunotherapies have revolutionized cancer care, but far from all patients could derive benefits from these therapies. In this study, we provided an integrated overview of tumor-infiltrating T cell profiles across multiple cancer types. We compared treatment-naive and post-ICB-treated samples, as well as ICB-R and ICB-NR samples, to identify pan-cancer immune correlates of ICB treatment. At a multidimensional level, we delved into the heterogeneity of T cells in the TME and the dynamic changes of immunotherapy at the pan-cancer level. We discovered global altering of T cell subtypes after patients received ICB treatment. Importantly, we categorized patients based on their response information. We deeply characterized the T cell landscape of pan-cancer response to tumor immunotherapy and answered the mechanisms underlying this pattern. Through the transcriptomic atlas, we discovered IRATs at the pan-cancer level. $CD4^+$ TGF-β1+ T and $CD8^+$ Temra cells were negatively correlated with response, while CXCL13+ T ($CD4^+$/$CD8^+$) cells were positively correlated with ICB treatment response. We found that at the pseudotime level, the two ICB treatment-responsive cell types differentiated earlier in the responders, while the two ICB treatment-resistant cell types showed opposing trends. This suggested that immunotherapy may reshape the TME by changing both proportion and differentiation time of the key T cell subtypes. We have also conducted in-depth studies on these four types of cells in terms of TF regulations and their cell-cell interaction patterns, describing the differences between the responsive and resistance groups at a multidimensional level.

In the subsequent immune atlas, we identified response-specific TCRs. TCR clonal expansion and selection are critical for generating TCR clones that recognize specific antigens expressed by the tumor cells, and thus identifying response-specific TCRs is essential for developing potential therapeutic targets. In all cancer types, the immune-profiling and transcriptomic patterns of these IRATs shared the same trend. The TCRs of $CD4^+$ TGF-β1+ T and $CD8^+$ Temra cells were significantly expanded in the non-responders, while the response-specific TCRs of CXCL13+ T cells demonstrated higher clonal expansion frequency in the responders than in the non-responders. This phenomenon emphasized the relationship between these IRATs with immune response and indicated the synergy and consistency of these T cells in both transcriptome and immune perspectives. Subsequently, we achieved the successful development of an index

that effectively discriminates treatment responsiveness. This index incorporates the transcriptomics and immune profiles of the IRATs, allowing for a comprehensive assessment of the patient's immune status. By integrating these factors, we established a robust framework that provides reliable predictions of treatment response. This index has significant implications for guiding therapeutic decisions and has the potential to greatly improve patient outcomes, particularly in the realm of immune-based therapies. Healthcare professionals can utilize this index to tailor treatment plans and optimize the effectiveness of immunotherapies, ultimately leading to better clinical outcomes and enhanced patient care.

In our third cohort, our main objective was to validate the presence of the IRATs in post-ICB-treated patients with CRC translationally. We accomplished this by employing single-cell proteomics analysis on patient PBMCs. Interestingly, we were able to confirm the expression of these IRATs in circulating blood, with similar proportions and trajectory patterns as observed in the local tissue except for undifferentiated trajectory patterns between ICB-Rs and ICB-NRs for $CD4^+$ CXCL13+ Tfh cells.

Among these IRATs, many studies have highlighted TRTs CXCL13+ T cells ($CD4^+$/$CD8^+$) as targets for cancer immunotherapy due to their association with improved clinical outcomes in many cancers.[22,26,29] Our research in the three cohorts confirmed that the presence of CXCL13+ T cells is a strong determinant of ICB response and could be served as potential targets for improving ICB response, not only in the first reported CRC multiomics dataset but also in a broader pan-cancer context. On the other hand, TNRTs $CD4^+$ TGF-β1+ T and $CD8^+$ Temra cells are shown in our study to contribute significantly to ICB non-responsiveness mechanistically in many molecular aspects. $CD4^+$ T cells expressing TGF-β have long been recognized to negatively impact tumor immunotherapy response.[49–53] However, recent clinical trials targeting both TGF-β and PD-1 have failed in phase 3 (NCT03631706),[54] which caused a great sensation. It is worth noting that TGF-β has three subtypes,[55] and only the TGF-β1 subtype may be effective when used in combination with immunotherapy.[55] Through our big data analysis, we identified a group of cells expressing only TGF-β1 but not TGF-β2/3, which played a role in immunotherapy resistance. Our findings suggested that the development of TGF-β1-specific antibodies could be a potential targeted therapy for patients with cancer. Temra cells, which are terminally differentiated effector memory cells, may also serve as a marker for immune non-responsiveness and a potential new target for treatment,[38,56] although more research is needed in this area.

Overall, this pan-cancer multiomics study highlights associations between T cell subsets and immunotherapy responsiveness and non-responsiveness and suggested that specific subsets may play important roles in the immunotherapy resistance. The insights and resources presented in this study may facilitate the identification of new biomarkers in clinical applications for disease diagnosis and treatments that target specific T cell populations.

Limitations of the study

While our study provides valuable insights into T cell dynamics and their role in ICB therapy response across multiple cancer types, several limitations warrant consideration. In terms of methodologies, the use of a single computational pipeline for batch correction and data integration may limit the generalizability of our findings to the current pipeline, and results might vary where different datasets or pipelines are used. Additionally, our study does not address the temporal changes in the TME that may influence ICB efficacy over the course of therapy. Longitudinal studies that track immune cell dynamics during treatment could offer more comprehensive insights into the evolution of immune responses. While we identify T cell subsets associated with immunotherapy response, future research should be conducted to confirm these findings through functional assays, including *in vivo* studies, to determine whether these T cell populations are drivers of resistance or merely associated epiphenomena.

RESOURCE AVAILABILITY

Lead contact

Further information and requests for resources and reagents should be directed to and will be fulfilled by the lead contact, Xuexin Li (xuexin.li@ki.se).

Materials availability

This study did not generate new unique reagents.

Data and code availability

- Both the raw and processed scRNA-seq, scImmune profiling, and CyTOF have been deposited in the National Genomics Data Center under the accession number PRJCA016919.
- All other data are available in the main text or in the supplementary materials.
- Source codes can be found in the GitHub repository under: https://github.com/pancancer/T.
- Any additional information required to reanalyze the data reported in this work paper is available from the lead contact upon request.

ACKNOWLEDGMENTS

This work was supported by grants from the National Natural Science Foundation of China 81860499, 82473450, and 32370713, Guangdong Basic and Applied Basic Research Foundation 2021A1515220145, International Science and Technology Cooperation Program of Liaoning Province 2024JH2/101900008, Public Health Research and Development Technology Special of Shenyang 81701699, the Joint Special Funds for the Department of Science and Technology of Yunnan Province Kunming Medical University 202401AY070001-344, and the Yunnan Major Scientific and Technological Projects 202302AA310044. We gratefully acknowledge the donors for their generous tissue donations to this project. We would also like to thank Uppsala University for providing resources and support through UPPMAX, which enabled us to conduct the computations and data handling for this project.

AUTHOR CONTRIBUTIONS

Conceptualization, X.L., R.S., Z.W., and Xiaolu Zhang; methodology, X.L., L.P., W.L., B.L., and Xuan Zhang; investigation, X.L., L.P., W.L., Xiaolu Zhang, F.G., and V.C.; analysis and visualization, L.P. and X.L.; funding acquisition, X.L., C.X., W.L., and Xiaolu Zhang; project administration, X.L., W.L., C.X., and Xiaolu Zhang; supervision, Z.W., R.S., and X.L.; writing – original draft, L.P., X.L., and W.L.; writing – review and editing, all authors.

DECLARATION OF INTERESTS

Authors declare that they have no competing interests.

CellPress
OPEN ACCESS

STAR★METHODS

Detailed methods are provided in the online version of this paper and include the following:

- KEY RESOURCES TABLE
- EXPERIMENTAL MODEL AND STUDY PARTICIPANT DETAILS
  - Participants
- METHOD DETAILS
  - Patient recruitment critieria and ethical approval
  - Treatment and follow-up
  - Fresh tissue dissociation and single-cell suspension preparation
  - Single-cell RNA-Sequencing
  - Mass cytometry
  - ScRNA-seq data processing and quality control
  - Integration, dimension reduction and clustering
  - Differential expression analysis
  - Cell type identification
  - Comparison of cell type proportions
  - GSEA, GO, and KEGG pathway analyses
  - Trajectory and RNA velocity analysis
  - Gene regulatory network analysis
  - Cell-cell communication
  - Multimodal TCR analysis and the identification of response-specific TCRs
  - Construction of response prediction model and response index (RI)
  - Survival analysis based on bulk RNA-Seq data
- QUANTIFICATION AND STATISTICAL ANALYSIS

SUPPLEMENTAL INFORMATION

Supplemental information can be found online at https://doi.org/10.1016/j.xcrm.2025.101992.

Received: August 3, 2024
Revised: January 15, 2025
Accepted: February 5, 2025
Published: March 6, 2025

REFERENCES

1. Anderson, N.M., and Simon, M.C. (2020). The tumor microenvironment. Curr. Biol. *30*, R921–R925. https://doi.org/10.1016/j.cub.2020.06.081.
2. Bottino, C., Picant, V., Vivier, E., and Castriconi, R. (2024). Natural killer cells and engagers: powerful weapons against cancer. Immunol. Rev. *328*, 412–421. https://doi.org/10.1111/imr.13384.
3. Tay, C., Tanaka, A., and Sakaguchi, S. (2023). Tumor-infiltrating regulatory T cells as targets of cancer immunotherapy. Cancer Cell *41*, 450–465. https://doi.org/10.1016/j.ccell.2023.02.014.
4. Korman, A.J., Garrett-Thomson, S.C., and Lonberg, N. (2022). The foundations of immune checkpoint blockade and the ipilimumab approval decennial. Nat. Rev. Drug Discov. *21*, 509–528. https://doi.org/10.1038/s41573-021-00345-8.
5. Freeman, A.J., Kearney, C.J., Silke, J., and Oliaro, J. (2021). Unleashing TNF cytotoxicity to enhance cancer immunotherapy. Trends Immunol. *42*, 1128–1142. https://doi.org/10.1016/j.it.2021.10.003.
6. Waldman, A.D., Fritz, J.M., and Lenardo, M.J. (2020). A guide to cancer immunotherapy: from T cell basic science to clinical practice. Nat. Rev. Immunol. *20*, 651–668. https://doi.org/10.1038/s41577-020-0306-5.
7. Sharma, P., Hu-Lieskovan, S., Wargo, J.A., and Ribas, A. (2017). Primary, Adaptive, and Acquired Resistance to Cancer Immunotherapy. Cell *168*, 707–723. https://doi.org/10.1016/j.cell.2017.01.017.
8. Li, J., Byrne, K.T., Yan, F., Yamazoe, T., Chen, Z., Baslan, T., Richman, L.P., Lin, J.H., Sun, Y.H., Rech, A.J., et al. (2018). Tumor Cell-Intrinsic Factors Underlie Heterogeneity of Immune Cell Infiltration and Response to Immunotherapy. Immunity *49*, 178–193.e7. https://doi.org/10.1016/j.immuni.2018.06.006.
9. O'Donnell, J.S., Teng, M.W.L., and Smyth, M.J. (2019). Cancer immunoediting and resistance to T cell-based immunotherapy. Nat. Rev. Clin. Oncol. *16*, 151–167. https://doi.org/10.1038/s41571-018-0142-8.
10. Pauken, K.E., Sammons, M.A., Odorizzi, P.M., Manne, S., Godec, J., Khan, O., Drake, A.M., Chen, Z., Sen, D.R., Kurachi, M., et al. (2016). Epigenetic stability of exhausted T cells limits durability of reinvigoration by PD-1 blockade. Science *354*, 1160–1165. https://doi.org/10.1126/science.aaf2807.
11. Sen, D.R., Kaminski, J., Barnitz, R.A., Kurachi, M., Gerdemann, U., Yates, K.B., Tsao, H.W., Godec, J., LaFleur, M.W., Brown, F.D., et al. (2016). The epigenetic landscape of T cell exhaustion. Science *354*, 1165–1169. https://doi.org/10.1126/science.aae0491.
12. Restifo, N.P., Smyth, M.J., and Snyder, A. (2016). Acquired resistance to immunotherapy and future challenges. Nat. Rev. Cancer *16*, 121–126. https://doi.org/10.1038/nrc.2016.2.
13. Jackson, C.M., Choi, J., and Lim, M. (2019). Mechanisms of immunotherapy resistance: lessons from glioblastoma. Nat. Immunol. *20*, 1100–1109. https://doi.org/10.1038/s41590-019-0433-y.
14. Rizvi, N.A., Hellmann, M.D., Snyder, A., Kvistborg, P., Makarov, V., Havel, J.J., Lee, W., Yuan, J., Wong, P., Ho, T.S., et al. (2015). Cancer immunology. Mutational landscape determines sensitivity to PD-1 blockade in non-small cell lung cancer. Science *348*, 124–128. https://doi.org/10.1126/science.aaa1348.
15. Yarchoan, M., Hopkins, A., and Jaffee, E.M. (2017). Tumor Mutational Burden and Response Rate to PD-1 Inhibition. N. Engl. J. Med. *377*, 2500–2501. https://doi.org/10.1056/NEJMc1713444.
16. Zheng, L., Qin, S., Si, W., Wang, A., Xing, B., Gao, R., Ren, X., Wang, L., Wu, X., Zhang, J., et al. (2021). Pan-cancer single-cell landscape of tumor-infiltrating T cells. Science *374*, abe6474. https://doi.org/10.1126/science.abe6474.
17. Bi, K., He, M.X., Bakouny, Z., Kanodia, A., Napolitano, S., Wu, J., Grimaldi, G., Braun, D.A., Cuoco, M.S., Mayorga, A., et al. (2021). Tumor and immune reprogramming during immunotherapy in advanced renal cell carcinoma. Cancer Cell *39*, 649–661.e5. https://doi.org/10.1016/j.ccell.2021.02.015.
18. Beltra, J.C., Manne, S., Abdel-Hakeem, M.S., Kurachi, M., Giles, J.R., Chen, Z., Casella, V., Ngiow, S.F., Khan, O., Huang, Y.J., et al. (2020). Developmental relationships of four exhausted CD8+ T cell subsets reveals underlying transcriptional and epigenetic landscape control mechanisms. Immunity *52*, 825–841.e8. https://doi.org/10.1016/j.immuni.2020.04.014.
19. Naulaerts, S., Datsi, A., Borras, D.M., Antoranz Martinez, A., Messiaen, J., Vanmeerbeek, I., Sprooten, J., Laureano, R.S., Govaerts, J., Panovska, D., et al. (2023). Multiomics and spatial mapping characterizes human CD8+ T cell states in cancer. Sci. Transl. Med. *15*, eadd1016. https://doi.org/10.1126/scitranslmed.add1016.
20. Paijens, S.T., Vledder, A., de Bruyn, M., and Nijman, H.W. (2021). Tumor-infiltrating lymphocytes in the immunotherapy era. Cell. Mol. Immunol. *18*, 842–859. https://doi.org/10.1038/s41423-020-00565-9.
21. Riaz, N., Havel, J.J., Makarov, V., Desrichard, A., Urba, W.J., Sims, J.S., Hodi, F.S., Martín-Algarra, S., Mandal, R., Sharfman, W.H., et al. (2017). Tumor and Microenvironment Evolution during Immunotherapy with Nivolumab. Cell *171*, 934–949.e16. https://doi.org/10.1016/j.cell.2017.09.028.
22. Mark, S., Karimi, E., Rezanejad, M., Yu, M.W., Desharnais, L., McDowell, S.A.C., Dore, S., Arabzadeh, A., Breton, V., Fiset, B., et al. (2023). Single-cell spatial landscape of immunotherapy response reveals mechanisms of CXCL13 enhanced antitumor immunity. J. ImmunoTher. Cancer *11*, e005545. https://doi.org/10.1136/jitc-2022-005545.
23. Tietscher, S., Wagner, J., Anzeneder, T., Langwieder, C., Rees, M., Sobottka, B., de Souza, N., and Bodenmiller, B. (2023). A comprehensive single-cell map of T cell exhaustion-associated immune environments in

human breast cancer. Nat. Commun. *14*, 98. https://doi.org/10.1038/s41467-022-35238-w.

24. Thommen, D.S., Koelzer, V.H., Herzig, P., Roller, A., Trefny, M., Dimeloe, S., Kiialainen, A., Hanhart, J., Schill, C., Hess, C., et al. (2018). A transcriptionally and functionally distinct PD-1+ CD8+ T cell pool with predictive potential in non-small-cell lung cancer treated with PD-1 blockade. Nat. Med. *24*, 994–1004. https://doi.org/10.1038/s41591-018-0057-z.

25. Yang, M., Lu, J., Zhang, G., Wang, Y., He, M., Xu, Q., Xu, C., and Liu, H. (2021). CXCL13 shapes immunoactive tumor microenvironment and enhances the efficacy of PD-1 checkpoint blockade in high-grade serous ovarian cancer. J. ImmunoTher. Cancer *9*, e001136. https://doi.org/10.1136/jitc-2020-001136.

26. Liu, B., Zhang, Y., Wang, D., Hu, X., and Zhang, Z. (2022). Single-cell meta-analyses reveal responses of tumor-reactive CXCL13+ T cells to immune-checkpoint blockade. Nat. Can. (Ott.) *3*, 1123–1136. https://doi.org/10.1038/s43018-022-00433-7.

27. Litchfield, K., Reading, J.L., Puttick, C., Thakkar, K., Abbosh, C., Bentham, R., Watkins, T.B.K., Rosenthal, R., Biswas, D., Rowan, A., et al. (2021). Meta-analysis of tumor- and T cell-intrinsic mechanisms of sensitization to checkpoint inhibition. Cell *184*, 596–614.e14. https://doi.org/10.1016/j.cell.2021.01.002.

28. Wang, F., Long, J., Li, L., Wu, Z.X., Da, T.T., Wang, X.Q., Huang, C., Jiang, Y.H., Yao, X.Q., Ma, H.Q., et al. (2023). Single-cell and spatial transcriptome analysis reveals the cellular heterogeneity of liver metastatic colorectal cancer. Sci. Adv. *9*, eadf5464. https://doi.org/10.1126/sciadv.adf5464.

29. Dai, S., Zeng, H., Liu, Z., Jin, K., Jiang, W., Wang, Z., Lin, Z., Xiong, Y., Wang, J., Chang, Y., et al. (2021). Intratumoral CXCL13+CD8+T cell infiltration determines poor clinical outcomes and immunoevasive contexture in patients with clear cell renal cell carcinoma. J. ImmunoTher. Cancer *9*, e001823. https://doi.org/10.1136/jitc-2020-001823.

30. Aoki, T., Chong, L.C., Takata, K., Milne, K., Marshall, A., Chavez, E.A., Miyata-Takata, T., Ben-Neriah, S., Unrau, D., Telenius, A., et al. (2021). Single-cell profiling reveals the importance of CXCL13/CXCR5 axis biology in lymphocyte-rich classic Hodgkin lymphoma. Proc. Natl. Acad. Sci. USA *118*, e2105822118. https://doi.org/10.1073/pnas.2105822118.

31. Workel, H.H., Lubbers, J.M., Arnold, R., Prins, T.M., van der Vlies, P., de Lange, K., Bosse, T., van Gool, I.C., Eggink, F.A., Wouters, M.C.A., et al. (2019). A Transcriptionally Distinct CXCL13+CD103+CD8+ T-cell Population Is Associated with B-cell Recruitment and Neoantigen Load in Human Cancer. Cancer Immunol. Res. *7*, 784–796. https://doi.org/10.1158/2326-6066.CIR-18-0517.

32. Batlle, E., and Massagué, J. (2019). Transforming Growth Factor-β Signaling in Immunity and Cancer. Immunity *50*, 924–940. https://doi.org/10.1016/j.immuni.2019.03.024.

33. Komuro, H., Shinohara, S., Fukushima, Y., Demachi-Okamura, A., Muraoka, D., Masago, K., Matsui, T., Sugita, Y., Takahashi, Y., Nishida, R., et al. (2023). Single-cell sequencing on CD8+ TILs revealed the nature of exhausted T cells recognizing neoantigen and cancer/testis antigen in non-small cell lung cancer. J. ImmunoTher. Cancer *11*, e007180. https://doi.org/10.1136/jitc-2023-007180.

34. Schalck, A., Sakellariou-Thompson, D., Forget, M.A., Sei, E., Hughes, T.G., Reuben, A., Bai, S., Hu, M., Kumar, T., Hurd, M.W., et al. (2022). Single-Cell Sequencing Reveals Trajectory of Tumor-Infiltrating Lymphocyte States in Pancreatic Cancer. Cancer Discov. *12*, 2330–2349. https://doi.org/10.1158/2159-8290.CD-21-1248.

35. Zhang, L., Yu, X., Zheng, L., Zhang, Y., Li, Y., Fang, Q., Gao, R., Kang, B., Zhang, Q., Huang, J.Y., et al. (2018). Lineage tracking reveals dynamic relationships of T cells in colorectal cancer. Nature *564*, 268–272. https://doi.org/10.1038/s41586-018-0694-x.

36. Eisenhauer, E.A., Therasse, P., Bogaerts, J., Schwartz, L.H., Sargent, D., Ford, R., Dancey, J., Arbuck, S., Gwyther, S., Mooney, M., et al. (2009). New response evaluation criteria in solid tumours: revised RECIST guideline (version 1.1). Eur. J. Cancer *45*, 228–247. https://doi.org/10.1016/j.ejca.2008.10.026.

37. Korsunsky, I., Millard, N., Fan, J., Slowikowski, K., Zhang, F., Wei, K., Baglaenko, Y., Brenner, M., Loh, P.R., and Raychaudhuri, S. (2019). Fast, sensitive and accurate integration of single-cell data with Harmony. Nat. Methods *16*, 1289–1296. https://doi.org/10.1038/s41592-019-0619-0.

38. Wistuba-Hamprecht, K., Martens, A., Heubach, F., Romano, E., Geukes Foppen, M., Yuan, J., Postow, M., Wong, P., Mallardo, D., Schilling, B., et al. (2017). Peripheral CD8 effector-memory type 1 T-cells correlate with outcome in ipilimumab-treated stage IV melanoma patients. Eur. J. Cancer *73*, 61–70. https://doi.org/10.1016/j.ejca.2016.12.011.

39. Aibar, S., González-Blas, C.B., Moerman, T., Huynh-Thu, V.,A., Imrichova, H., Hulselmans, G., Rambow, F., Marine, J.C., Geurts, P., Aerts, J., et al. (2017). SCENIC: single-cell regulatory network inference and clustering. Nat. Methods *14*, 1083–1086. https://doi.org/10.1038/nmeth.4463.

40. Qiu, X., Mao, Q., Tang, Y., Wang, L., Chawla, R., Pliner, H.A., and Trapnell, C. (2017). Reversed graph embedding resolves complex single-cell trajectories. Nat. Methods *14*, 979–982. https://doi.org/10.1038/nmeth.4402.

41. Efremova, M., Vento-Tormo, M., Teichmann, S.A., and Vento-Tormo, R. (2020). CellPhoneDB: inferring cell–cell communication from combined expression of multi-subunit ligand–receptor complexes. Nat. Protoc. *15*, 1484–1506. https://doi.org/10.1038/s41596-020-0292-x.

42. Bhat, P., Leggatt, G., Waterhouse, N., and Frazer, I.H. (2017). Interferon-γ derived from cytotoxic lymphocytes directly enhances their motility and cytotoxicity. Cell Death Dis. *8*, e2836. https://doi.org/10.1038/cddis.2017.67.

43. Ozga, A.J., Chow, M.T., and Luster, A.D. (2021). Chemokines and the immune response to cancer. Immunity *54*, 859–874. https://doi.org/10.1016/j.immuni.2021.01.012.

44. Liberzon, A., Subramanian, A., Pinchback, R., Thorvaldsdóttir, H., Tamayo, P., and Mesirov, J.P. (2011). Molecular signatures database (MSigDB) 3.0. Bioinformatics *27*, 1739–1740. https://doi.org/10.1093/bioinformatics/btr260.

45. Balar, A.V., Galsky, M.D., Rosenberg, J.E., Powles, T., Petrylak, D.P., Bellmunt, J., Loriot, Y., Necchi, A., Hoffman-Censits, J., Perez-Gracia, J.L., et al. (2017). Atezolizumab as first-line treatment in cisplatin-ineligible patients with locally advanced and metastatic urothelial carcinoma: a single-arm, multicentre, phase 2 trial. Lancet *389*, 67–76. https://doi.org/10.1016/S0140-6736(16)32455-2.

46. Braun, D.A., Hou, Y., Bakouny, Z., Ficial, M., Sant' Angelo, M., Forman, J., Ross-Macdonald, P., Berger, A.C., Jegede, O.A., Elagina, L., et al. (2020). Interplay of somatic alterations and immune infiltration modulates response to PD-1 blockade in advanced clear cell renal cell carcinoma. Nat. Med. *26*, 909–918. https://doi.org/10.1038/s41591-020-0839-y.

47. Liu, D., Schilling, B., Liu, D., Sucker, A., Livingstone, E., Jerby-Arnon, L., Zimmer, L., Gutzmer, R., Satzger, I., Loquai, C., et al. (2019). Integrative molecular and clinical modeling of clinical outcomes to PD1 blockade in patients with metastatic melanoma. Nat. Med. *25*, 1916–1927. https://doi.org/10.1038/s41591-019-0654-5.

48. Lausen, B., and Schumacher, M. (1992). Maximally selected rank statistics. Biometrics *48*, 73–85. https://doi.org/10.2307/2532740.

49. Derynck, R., Turley, S.J., and Akhurst, R.J. (2021). TGFβ biology in cancer progression and immunotherapy. Nat. Rev. Clin. Oncol. *18*, 9–34. https://doi.org/10.1038/s41571-020-0403-1.

50. Li, S., Liu, M., Do, M.H., Chou, C., Stamatiades, E.G., Nixon, B.G., Shi, W., Zhang, X., Li, P., Gao, S., et al. (2020). Cancer immunotherapy via targeted TGF-β signalling blockade in TH cells. Nature *587*, 121–125. https://doi.org/10.1038/s41586-020-2850-3.

51. Chen, S.Y., Mamai, O., and Akhurst, R.J. (2022). TGFβ: Signaling Blockade for Cancer Immunotherapy. Annu. Rev. Cell Biol. *6*, 123–146. https://doi.org/10.1146/annurev-cancerbio-070620-103554.

52. Holmgaard, R.B., Schaer, D.A., Li, Y., Castaneda, S.P., Murphy, M.Y., Xu, X., Inigo, I., Dobkin, J., Manro, J.R., Iversen, P.W., et al. (2018). Targeting

the TGFβ pathway with galunisertib, a TGFβRI small molecule inhibitor, promotes anti-tumor immunity leading to durable, complete responses, as monotherapy and in combination with checkpoint blockade. J. ImmunoTher. Cancer *6*, 47. https://doi.org/10.1186/s40425-018-0356.4.

53. Groeneveldt, C., van Ginkel, J.Q., Kinderman, P., Sluijter, M., Griffioen, L., Labrie, C., van den Wollenberg, D.J.M., Hoeben, R.C., van der Burg, S.H., Ten Dijke, P., et al. (2023). Intertumoral Differences Dictate the Outcome of TGF-β Blockade on the Efficacy of Viro-Immunotherapy. Cancer Res. Commun. *3*, 325–337. https://doi.org/10.1158/2767-9764.CRC-23-0019.

54. Metropulos, A.E., Munshi, H.G., and Principe, D.R. (2022). The difficulty in translating the preclinical success of combined TGFβ and immune checkpoint inhibition to clinical trial. EBioMedicine *86*, 104380. https://doi.org/10.1016/j.ebiom.2022.104380.

55. Martin, C.J., Datta, A., Littlefield, C., Kalra, A., Chapron, C., Wawersik, S., Dagbay, K.B., Brueckner, C.T., Nikiforov, A., Danehy, F.T., Jr., et al. (2020). Selective inhibition of TGFβ1 activation overcomes primary resistance to checkpoint blockade therapy by altering tumor immune landscape. Sci. Transl. Med. *12*, eaay8456. https://doi.org/10.1126/scitranslmed.aay8456.

56. Lee, S.W., Choi, H.Y., Lee, G.W., Kim, T., Cho, H.J., Oh, I.J., Song, S.Y., Yang, D.H., and Cho, J.H. (2021). CD8(+) TILs in NSCLC differentiate into TEMRA via a bifurcated trajectory: deciphering immunogenicity of tumor antigens. J. Immunother. Cancer *9*, e002709. https://doi.org/10.1136/jitc-2021-002709.

57. Yost, K.E., Satpathy, A.T., Wells, D.K., Qi, Y., Wang, C., Kageyama, R., McNamara, K.L., Granja, J.M., Sarin, K.Y., Brown, R.A., et al. (2019). Clonal replacement of tumor-specific T cells following PD-1 blockade. Nat. Med. *25*, 1251–1259. https://doi.org/10.1038/s41591-019-0522-3.

58. Bassez, A., Vos, H., Van Dyck, L., Floris, G., Arijs, I., Desmedt, C., Boeckx, B., Vanden Bempt, M., Nevelsteen, I., Lambein, K., et al. (2021). A single-cell map of intratumoral changes during anti-PD1 treatment of patients with breast cancer. Nat. Med. *27*, 820–832. https://doi.org/10.1038/s41591-021-01323-8.

59. Krishna, C., DiNatale, R.G., Kuo, F., Srivastava, R.M., Vuong, L., Chowell, D., Gupta, S., Vanderbilt, C., Purohit, T.A., Liu, M., et al. (2021). Single-cell sequencing links multiregional immune landscapes and tissue-resident T cells in ccRCC to tumor topology and therapy efficacy. Cancer Cell *39*, 662–677.e6. https://doi.org/10.1016/j.ccell.2021.03.007.

60. Ma, L., Wang, L., Khatib, S.A., Chang, C.W., Heinrich, S., Dominguez, D.A., Forgues, M., Candia, J., Hernandez, M.O., Kelly, M., et al. (2021). Single-cell atlas of tumor cell evolution in response to therapy in hepatocellular carcinoma and intrahepatic cholangiocarcinoma. J. Hepatol. *75*, 1397–1408. https://doi.org/10.1016/j.jhep.2021.06.028.

61. Luoma, A.M., Suo, S., Wang, Y., Gunasti, L., Porter, C.B.M., Nabilsi, N., Tadros, J., Ferretti, A.P., Liao, S., Gurer, C., et al. (2022). Tissue-resident memory and circulating T cells are early responders to pre-surgical cancer immunotherapy. Cell *185*, 2918–2935.e29. https://doi.org/10.1016/j.cell.2022.06.018.

62. Oh, D.Y., Kwek, S.S., Raju, S.S., Li, T., McCarthy, E., Chow, E., Aran, D., Ilano, A., Pai, C.C.S., Rancan, C., et al. (2020). Intratumoral CD4+ T Cells Mediate Anti-tumor Cytotoxicity in Human Bladder Cancer. Cell *181*, 1612–1625.e13. https://doi.org/10.1016/j.cell.2020.05.017.

63. Caushi, J.X., Zhang, J., Ji, Z., Vaghasia, A., Zhang, B., Hsiue, E.H.C., Mog, B.J., Hou, W., Justesen, S., Blosser, R., et al. (2021). Transcriptional programs of neoantigen-specific TIL in anti-PD-1-treated lung cancers. Nature *596*, 126–132. https://doi.org/10.1038/s41586-021-03752-4.

64. Wu, T.D., Madireddi, S., de Almeida, P.E., Banchereau, R., Chen, Y.J.J., Chitre, A.S., Chiang, E.Y., Iftikhar, H., O'Gorman, W.E., Au-Yeung, A., et al. (2020). Peripheral T cell expansion predicts tumour infiltration and clinical response. Nature *579*, 274–278. https://doi.org/10.1038/s41586-020-2056-8.

65. He, S., Wang, L.H., Liu, Y., Li, Y.Q., Chen, H.T., Xu, J.H., Peng, W., Lin, G.W., Wei, P.P., Li, B., et al. (2020). Single-cell transcriptome profiling of an adult human cell atlas of 15 major organs. Genome Biol. *21*, 294. https://doi.org/10.1186/s13059-020-02210-0.

66. Luoma, A.M., Suo, S., Williams, H.L., Sharova, T., Sullivan, K., Manos, M., Bowling, P., Hodi, F.S., Rahma, O., Sullivan, R.J., et al. (2020). Molecular Pathways of Colon Inflammation Induced by Cancer Immunotherapy. Cell *182*, 655–671.e22. https://doi.org/10.1016/j.cell.2020.06.001.

67. Corridoni, D., Antanaviciute, A., Gupta, T., Fawkner-Corbett, D., Aulicino, A., Jagielowicz, M., Parikh, K., Repapi, E., Taylor, S., Ishikawa, D., et al. (2020). Single-cell atlas of colonic CD8+ T cells in ulcerative colitis. Nat. Med. *26*, 1480–1490. https://doi.org/10.1038/s41591-020-1003-4.

68. Zunder, E.R., Finck, R., Behbehani, G.K., Amir, E.A.D., Krishnaswamy, S., Gonzalez, V.D., Lorang, C.G., Bjornson, Z., Spitzer, M.H., Bodenmiller, B., et al. (2015). Palladium-based mass tag cell barcoding with a doublet-filtering scheme and single-cell deconvolution algorithm. Nat. Protoc. *10*, 316–333. https://doi.org/10.1038/nprot.2015.020.

69. Finck, R., Simonds, E.F., Jager, A., Krishnaswamy, S., Sachs, K., Fantl, W., Pe'er, D., Nolan, G.P., and Bendall, S.C. (2013). Normalization of mass cytometry data with bead standards. Cytometry A. *83*, 483–494. https://doi.org/10.1002/cyto.a.22271.

70. Nowicka, M., Krieg, C., Crowell, H.L., Weber, L.M., Hartmann, F.J., Guglietta, S., Becher, B., Levesque, M.P., and Robinson, M.D. (2017). CyTOF workflow: differential discovery in high-throughput high-dimensional cytometry datasets. F1000Res. *6*, 748. https://doi.org/10.12688/f1000research.11622.3.eCollection2017.

71. Patil, I. (2021). Visualizations with statistical details: The'ggstatsplot'approach. J. Open Source Softw. *6*, 3167. https://doi.org/10.21105/joss.03167.

72. McGinnis, C.S., Murrow, L.M., and Gartner, Z.J. (2019). DoubletFinder: Doublet Detection in Single-Cell RNA Sequencing Data Using Artificial Nearest Neighbors. Cell Syst. *8*, 329–337.e4. https://doi.org/10.1016/j.cels.2019.03.003.

73. Hao, Y., Hao, S., Andersen-Nissen, E., Mauck, W.M., 3rd, Zheng, S., Butler, A., Lee, M.J., Wilk, A.J., Darby, C., Zager, M., et al. (2021). Integrated analysis of multimodal single-cell data. Cell *184*, 3573–3587.e29. https://doi.org/10.1016/j.cell.2021.04.048.

74. Becht, E., McInners, L., Healy, J., Dutertre, C.A., Kwok, I.W.H., Ng, L.G., Ginhoux, F., and Newell, E.W. (2018). Dimensionality reduction for visualizing single-cell data using UMAP. Nat. Biotechnol. *37*, 38–44. https://doi.org/10.1038/nbt.4314.

75. Domínguez Conde, C., Xu, C., Jarvis, L.B., Rainbow, D.B., Wells, S.B., Gomes, T., Howlett, S.K., Suchanek, O., Polanski, K., King, H.W., et al. (2022). Cross-tissue immune cell analysis reveals tissue-specific features in humans. Science *376*, eabl5197. https://doi.org/10.1126/science.abl5197.

76. Ritchie, M.E., Phipson, B., Wu, D., Hu, Y., Law, C.W., Shi, W., and Smyth, G.K. (2015). limma powers differential expression analyses for RNA-sequencing and microarray studies. Nucleic Acids Res. *43*, e47. https://doi.org/10.1093/nar/gkv007.

77. Cao, J., Spielmann, M., Qiu, X., Huang, X., Ibrahim, D.M., Hill, A.J., Zhang, F., Mundlos, S., Christiansen, L., Steemers, F.J., et al. (2019). The single-cell transcriptional landscape of mammalian organogenesis. Nature *566*, 496–502. https://doi.org/10.1038/s41586-019-0969-x.

78. Huynh-Thu, V.A., Irrthum, A., Wehenkel, L., and Geurts, P. (2010). Inferring Regulatory Networks from Expression Data Using Tree-Based Methods. PLoS One *5*, e12776. https://doi.org/10.1371/journal.pone.0012776.

79. Hou, R., Denisenko, E., Ong, H.T., Ramilowski, J.A., and Forrest, A.R.R. (2020). Predicting cell-to-cell communication networks using NATMI. Nat. Commun. *11*, 5011. https://doi.org/10.1038/s41467-020-18873-z.

80. Raredon, M.S.B., Yang, J., Garritano, J., Wang, M., Kushnir, D., Schupp, J.C., Adams, T.S., Greaney, A.M., Leiby, K.L., Kaminski, N., et al. (2022). Computation and visualization of cell-cell signaling topologies in single-cell systems data using Connectome. Sci. Rep. *12*, 4187. https://doi.org/10.1038/s41598-022-07959-x.

81. Cabello-Aguilar, S., Alame, M., Kon-Sun-Tack, F., Fau, C., Lacroix, M., and Colinge, J. (2020). SingleCellSignalR: inference of intercellular networks from single-cell transcriptomics. Nucleic Acids Res. *48*, e55. https://doi.org/10.1093/nar/gkaa183.

82. Dimitrov, D., Turei, D., Garrido-Rodriguez, M., Burmedi, P.L., Nagai, J.S., Boys, C., Flores, R.O.R., Kim, H., Szalai, B., Costa, I.G., et al. (2022). Comparison of methods and resources for cell-cell communication inference from single-cell RNA-Seq data. Nat. Commun. *13*, 3224. https://doi.org/10.1038/s41467-022-30755-0.

83. Türei, D., Valdeolivas, A., Gul, L., Palacio-Escat, N., Klein, M., Ivanova, O., Olbei, M., Gabor, A., Theis, F., Modos, D., et al. (2021). Integrated intra- and intercellular signaling knowledge for multicellular omics analysis. Mol. Syst. Biol. *17*, e9923. https://doi.org/10.15252/msb.20209923.

84. Borcherding, N., Bormann, N.L., and Kraus, G. (2020). scRepertoire: An R-based toolkit for single-cell immune receptor analysis. F1000Res. *9*, 47. https://doi.org/10.12688/f1000research.22139.2.eCollection2020.

85. Subramanian, A., Tamayo, P., Mootha, V.K., Mukherjee, S., Ebert, B.L., Gillette, M.A., Paulovich, A., Pomeroy, S.L., Golub, T.R., Lander, E.S., and Mesirov, J.P. (2005). Gene set enrichment analysis: a knowledge-based approach for interpreting genome-wide expression profiles. Proc. Natl. Acad. Sci. USA *102*, 15545–15550. https://doi.org/10.1073/pnas.0506580102.

86. de Jong, A., Kuipers, O.P., and Kok, J. (2022). FUNAGE-Pro: comprehensive web server for gene set enrichment analysis of prokaryotes. Nucleic Acids Res. *50*, W330–W336. https://doi.org/10.1093/nar/gkac441.

87. Engebretsen, S., and Bohlin, J. (2019). Statistical predictions with glmnet. Clin. Epigenet. *11*, 123. https://doi.org/10.1186/s13148-019-0730-1.

88. Hothorn, T., and Lausen, B. (2003). On the exact distribution of maximally selected rank statistics. Comput. Stat. Data Anal. *43*, 121–137. https://doi.org/10.1016/s0167-9473(02)00225-6.

STAR★METHODS

KEY RESOURCES TABLE

REAGENT or RESOURCE	SOURCE	IDENTIFIER
**Biological samples**		
Human CRC tumor tissue	This paper	N/A
Human normal colon tissue	This paper	N/A
Peripheral blood sample of CRC patients	This paper	N/A
**Deposited data**		
Sequencing data of BCC patients	GEO	GEO: GSE123813
Sequencing data of Breast Cancer patients	EGA	EGAD00001006608
Sequencing data of ccRCC patients	SRA	SRZ190804
Sequencing data of HCC patients	GEO	GEO: GSE151530
Sequencing data of HNSCC patients	GEO	GEO: GSE200996
Sequencing data of iCCA patients	GEO	GEO: GSE151530
Sequencing data of MIBC patients	GEO	GEO: GSE149652
Sequencing data of NSCLC patients	GEO	GEO: GSE176021
Sequencing data of SCC patients	GEO	GEO: GSE123813
Sequencing data of normal bladder	GEO	GEO: GSE159929
Sequencing data of normal colon	GEO	GEO: GSE144469
Sequencing data of normal kidney	GEO	GEO: GSE139555
Sequencing data of normal lung	GEO	GEO: GSE139555
Sequencing data of normal skin	GEO	GEO: GSE176021
**Software and algorithms**		
R Studio	The R Project for Statistical Computing	http://www.r-project.org/
Microsoft Excel for Mac	Microsoft	http://products.office.com/en-us/excel
Cellranger	10XGenomics v6.1.0	http://github.com/10XGenomics/cellranger
Seurat	v4.2.1	http://satijalab.org/seurat/articles/install.html
**Other**		
Raw and processed sequencing data of CRC patients	This paper	PRJCA016919
CyTOF data of CRC patients	This paper	PRJCA016919
Source code		https://github.com/pancancer/T

EXPERIMENTAL MODEL AND STUDY PARTICIPANT DETAILS

Participants

Thirty-seven newly-enrolled CRC patients and 3 donors were included in the current study, among which, peripheral blood samples were obtained from 17 CRC patients for CyTOF analysis; tumor tissues from 15 CRC patients (5 patients had both pre- and post-anti-PD1 treatment tumor samples, 4 patients only had treatment-naïve samples and 6 patients had post-*anti*-PD1 treatment samples) were processed for single-cell RNA sequencing; tumor tissue samples from 19 CRC patients (14 had scRNA transcriptomics data as well) and normal colon tissues from 3 donors were processed for single-cell Immune sequencing (see Tables S1 and S2 for details).

Besides, patients with deposited sequencing data were collected as well, including 15 NSCLC patients, 11 HCC patients, 8 iCCA patients, 6 MIBC patients, 19 HNSCC patients, 31 breast cancer patients, 9 ccRCC patients 8 BCC patients and 4 SCC patients (see Table S1 for details).

METHOD DETAILS

Patient recruitment critieria and ethical approval

The datasets of the omics were obtained from a combination of newly collected and previously published datasets (see Tables S1 and S2 for details).[17,57–67] The new datasets comprised of CRC patients enrolled with the following criteria: (i) CRC stage II (cT3-4,

N0)/III stage (any cT, N+); (ii) aged ≧18 years old with no gender limitations; (iii) histopathologically confirmed CRC of any pathological type; (iv) confirmed by sequencing/immunohistochemistry (IHC)/polymerase chain reaction (PCR) to have proficient mismatch repair (pMMR)/microsatellite stable (MSS) status, or deficient mismatch repair (dMMR)/microsatellite instability-high (MSI-H); (v) those who were immunotherapy-treated have received neoadjuvant PD-1 monotherapy; (vi) able to undergo colonoscopy biopsy with a body weight greater than or equal to 40kg and a life expectancy greater than or equal to 6 months. The study excluded individuals who received chemotherapy, targeted therapy, other combined treatment regimens, or Chinese patent medicines during the same cycle. Additionally, those who received other immunotherapy (PD-1, CTLA-4) regimens during the same cycle or any other antibodies that act on T cell co-stimulation or checkpoint pathways were also excluded. Based on the inclusion and exclusion criteria, we collected a total of 480 cancer samples from 10 cancer types and 27 samples from five normal tissue types, including samples from breast cancer, NSCLC, HNSCC, BCC, ccCRCC, CRC, MIBC, SCC, HCC, and iCCA. Among them, there were 244 scRNA-Seq samples collected, which included 83 ICB pre-treatment samples and 161 post-treatment samples with clinical response information; 240 scImmune-profiling samples, which included 213 cancer samples and 27 non-tumor samples, and of the 213 samples, 73 were ICB pre-treatment samples and 140 were post-treatment samples with clinical response information; and 23 CyTOF samples, which included 16 ICB pre-treatment samples and 7 post-treatment samples with clinical response information.

This study was conducted in accordance with the Declaration of Helsinki and was approved by the Institutional Review Board of Yunnan Cancer Center (approval number KYCS202199).

Treatment and follow-up

The decisions to treat patients with PD-1 inhibitors instead of surgery were made by the primary surgeons of the participating centers. The most common reason for this decision was to avoid multivisceral resection and sphincter dysfunction. As there was no guideline recommending PD-1 inhibitors as neoadjuvant treatment of CRC, the decisions were made based on empirical experience, resulting in non-unanimous regimens. Notably, the study used several kinds of PD-1 inhibitors, including pembrolizumab, sintilimab, camrelizumab, and tislelizumab. Radiographic assessment of response was generally performed after two cycles of treatment, and every two to three months thereafter before surgery (if performed), according to the revised RECIST guideline (version 1.1). For patients whose tumor had achieved clinical complete response (cCR; defined as the absence of tumor on radiologic and endoscopic findings), a watch-and-wait approach was offered according to clinical practice.

Fresh tissue dissociation and single-cell suspension preparation

The colorectal tissues were preserved using GEXSCOPE Tissue Preservation Solution and kept on ice. These samples were washed three times with Hanks' balanced salt solution (HBSS, Gibco, Cat. No.14025-076) and shredded into 1–2 mm pieces. The tissue debris was then subjected to digestion with 2 mL of GEXSCOPE tissue dissociation solution in a 15 mL centrifuge tube (Falcon, Cat. No.352095) with sustained agitation at 37°C for 15 min.

The digested tissues were filtered through a 100-micron filter and washed twice with PBS before surface staining. Anti-CD45 and DAPI were used for immune cell staining at 1×10^6 cells per mL for 20 min to discriminate live and dead cells after Fc receptor blockade (BioLegend). Viable immune cells ($CD45^+DAPI^-$) were then sorted and collected on a FACSARIA sorter (BD Biosciences).

$CD45^+$ cells were filtered through 40-micron sterile strainers (Falcon, Cat. No.352340) and centrifuged (Eppendorf, 5810R) at 300g for 5 min. The supernatant was removed, and the pellets were gently resuspended in 1mL PBS (Hyclone, Cat. No.SA30256.01). To remove red blood cells (RBC), which were frequently a significant portion of the cells produced, 2 mL RBC lysis buffer (Roche, Cat. No. 11 814 389 001) was added to the cell suspension following the manufacturer's protocol. The cells were then centrifuged at 500 × g for 5 min in a microfuge at 15°C–25°C and resuspended in PBS (Hyclone, Cat. No.SA30256.01). A trypan blue stain (Bio-RAD, Cat. No.#1450013) was used to count the cells under a microscope (Nikon, ECLIPSE Ts2), and the concentration was adjusted to 1×10^5 cells/mL. Subsequent sample processing was performed once the cell viability exceeded 80%.

Single-cell RNA-Sequencing

Cell capture and cDNA synthesis

Using single cell 5′Library and Gel Bead Kit (10x Genomics, 1000006) and Chromium Single Cell A Chip Kit (10x Genomics, 120236), the cell suspension (300–600 living cells per microliter determined by Count Star) was loaded onto the Chromium single cell controller (10x Genomics) to generate single-cell gel beads in the emulsion according to the manufacturer's protocol. In short, single cells were suspended in PBS containing 0.04% BSA. About 6,000 cells were added to each channel, and the target cell will be recovered was estimated to be about 3,000 cells. Captured cells were lysed and the released RNA were barcoded through reverse transcription in individual GEMs. Reverse transcription was performed on a S1000TM Touch Thermal Cycler (Bio Rad) at 53°C for 45 min, followed by 85°C for 5 min, and hold at 4°C. The cDNA was generated and then amplified, and quality assessed using an Agilent 4200 (performed by CapitalBio Technology, Beijing).

Library preparation

According to the manufacture's introduction, single-cell RNA-Seq libraries were constructed using Single Cell 5′ Library and Gel Bead Kit, Single Cell V(D)J Enrichment Kit, Human T cell (1000005) and Single Cell V(D)J Enrichment Kit, Human B Cell (1000016). The libraries were sequenced using Illumina Novaseq6000 sequencer with a sequencing depth of at least 100,000 reads per cell with pair-end 150 bp (PE150) reading strategy (performed by CapitalBio Technology, Beijing).

CellPress
OPEN ACCESS

Mass cytometry

Patient Samples Collection and mass cytometry preparation

Patients with CRC who were receiving anti-PD-1 immune checkpoint blockade (ICB) with pembrolizumab or sintilimab at Yunnan Cancer Center and Sun Yat-sen University Cancer Center were recruited for the study. These patients provided written informed consent and were enrolled in accordance with the Institutional Review Board guidelines of each institution. Patients received intravenous pembrolizumab or sintilimab (200 mg) every 3 weeks. Blood samples were collected at baseline and during patient PR for monitoring treatment response using the RECIST 1.1 criteria.

Antibodies labeling and optimization

For mass cytometry experiments, purified antibodies were obtained from BioLegend, Thermo Fisher, BioRAD and R&D systems. Antibody labeling with the indicated metal tag was performed using the MaxPAR antibody Labeling kit (Fluidigm). Conjugated antibodies were titrated for optimal concentration before use.

Sample stimulation, cell staining, and data acquisition

Cells were thawed and washed with 1xPBS and then stained with 100μL of 250nM cisplatin (Fluidigm) for 5min on ice to exclude dead cells, and then incubated in Fc receptor blocking solution before stained with surface antibodies cocktail for 30 min on ice. Cells were washed twice with FACS buffer (1xPBS+0.5%BSA) and fixed in 200μL of intercalation solution (Maxpar Fix and Perm Buffer containing 250nM 191/193Ir, Fluidigm) overnight. After fixation, cells were washed once with FACS buffer and then perm buffer (eBioscience), stained with intracellular antibodies cocktail for 30 min on ice. Cells were washed and resuspend with deionized water, adding into 20% EQ beads (Fluidigm), acquired on a mass cytometer (Helios, Fluidigm). For stimulation, freshly prepared 150 ng/ml of Phorbol 12-Myristate 13-Acetate (PMA; Cat No: P8139; Sigma-Aldrich, St. Louis, MO) and 100 ng/mL of iono-mycin (Cat No: 10634; Sigma-Aldrich, St. Louis, MO) were added and incubated at 37°C for 6h.

Data preprocessing and quality control

Data of each sample were debarcoded from raw data using a doublet-filtering scheme[68] with unique mass-tagged barcodes. Each *.fcs* file generated from different batches were normalized through bead normalization method.[69] Manual gating was done using FlowJo to exclude to debris, dead cells and doublets, leaving CD45 positive live immune cells. We have also demonstrated the gating quality of the CyTOF samples after stimulation via the gating of CXCL13+ $CD4^+$ T cells and Granzyme K+ $CD8^+$ T cells.

Data Analysis

Based on the CD45 positive live cells, dataset was first arcsinh-transformed with a cofactor of 5[70] to make large-ranged highly skewed CyTOF data to an approximated normal distribution. Batch effect was assessed and corrected using the soft-clustering approach.[37] This was followed by dimension reduction based on uniform manifold approximation and projection (UMAP) and unsupervised meta-clustering to split the cells into $CD3^+CD4^+CD8^-$, $CD3^+CD8^+CD4^-$, and the other cells using Seurat. Based on the $CD4^+$ and $CD8^+$ T cells, we separately performed clustering to further dissect these T cells into small populations. For each population of cells, differential expression (DE) analysis was performed using the two-tailed Wilcoxon Rank-Sum test to compare each population of cells with all the other cells. Bonferrroni correction was done to correct for multiple testing. Markers with Bonferroni-adjusted $p < 0.05$ were retained as differentially expressed genes (DEGs) for each cell cluster. We discovered nine $CD4^+$ T cell types and eight $CD8^+$ T cell types based on their marker expressions in the clusters. To compare the proportions between the R and the NR groups, based on the findings, for each cell type, we performed two-proportions z-test between the R and the NR group to examine whether the cell type proportion were equal between the two groups. In pseudotime analysis, for each cell type in each response group, we ordered the cells based on ranked PC1 coordinates as a pseudotime basis. In order to compare the pseudotime between the groups, we scaled the pseudotime into a range of 0–1 and compared statistically the distributions of the scaled pseudotime in each cell type of each response group using the Welch's t-test.[71]

ScRNA-seq data processing and quality control

For scRNA-Seq, Cellranger v6.1.0 were used for de-multiplexing, mapping, and quantification after sequencing. Human genome reference GRCh38 was used for read alignment and quantification was done using the *count* function from Cellranger to obtain gene-cell count matrix for each sample. The gene-cell count matrix of each sample was subjected to quality control assessment, and cells with less than 200 or more than 6000 detected genes were filtered out. To remove potential debris and damaged cells from affecting the overall quality of the data, we assessed the percentage of mitochondrial reads sequenced per cell and removed cells with >30% mitochondrial reads. In addition, DoubletFinder[72] was used to remove potential doublets present in the dataset. To maximized the accuracy of identifying doublets, an optimal principal component neighborhood size (pK) was chosen based on the highest bimodality coefficient (BC) measure for each sample, using the mean-variance-normalized bimodality coefficient (BC_{MVN}) maximization[72] strategy.

For scRNA-Seq datasets collected from published studies, gene-cell count matrices, cell type annotations, clinical and sample metadata were obtained from the data repositories hosting these publication data. For unprocessed datasets, the same set of quality control assessments were conducted as described above, except that the filtering stringency was enhanced for these public data in terms percentage of mitochondrial reads. To obtain a high quality of the public dataset, cells with >10% mitochondrial reads were removed.

Count data from both the inhouse dataset as well as previously published datasets were normalized with a scale factor of 10,000, and natural-log transformed using Seurat v4.2.1.[73] Normalized data were scaled and centered. Using the expression counts

standardized based on observed mean and variance in the fitted log-variance to log-mean locally estimated scatterplot smoothing regression model, genes with the highest variance (top n = 2000) were chosen to perform a principal component analysis (PCA) dimension reduction.

Integration, dimension reduction and clustering

For inhouse and published scRNA-Seq datasets, degree of variabilities was assessed to consider for integration. Each study dataset was treated as a separate batch to makes sure that the inter-study variabilities were addressed. Integration was performed using Harmony v0.1.1,[37] and based on the first 30 PCA coordinates, k-means soft-clustering was performed to classify cells into clusters. Squared Euclidean distance between PCA embeddings (Z) of cells and their centroids (Y) was computed as follows,

$$\min_{R,Y} \sum_{i,k} R_{ki} 2\left(1 - Y_k^T Z_i\right) + \sigma R_{ki} \log R_{ki} + \sigma \theta R_{ki} \log\left(\frac{O_{ki}}{E_{ki}}\right) \phi_i$$

$$s.t.\ \forall_i \forall_k R_{ki} > 0,\ \forall_i \sum_{k=1}^{K} R_{ki} = 1$$

where ϕ denotes the batch assignment for each cell i, and R represents cluster assignment matrix with each cluster identified as k.[37] For each batch of cells in each cluster k, a centroid was computed, and a correction factor was determined for each batch to correct for batch differences within the 2-dimensional orientation of the cells based on their soft cluster assignments. A corrected set of PCA embeddings, named the harmony embeddings were returned after integration. The quality of integration was assessed by the PCA-corrected harmony embeddings.

Using the first 30 harmony embeddings, UMAP dimension reduction was performed using Seurat v4.2.1.[73,74] The Jaccard index between each cell and its nearest neighbors (k = 20) was calculated to obtain a shared neighbor graph (SNN) for the integrated data. SNN modularity optimization was performed to determine the clustering assignment for each cell.[73]

Differential expression analysis

DE analysis was performed using two-tailed Wilcoxon Rank-Sum test to compare each cluster of cells with the remaining cells.[73] BH FDR correction was done to correct for multiple testing. Genes with FDR <0.05 were retained as differentially expressed genes (DEGs) for each cell cluster.

Cell type identification

Cell type annotation was performed in three separate steps. First, for published datasets used, the cell annotations provided by the publications were served as reference to the current cell annotations. For in-house dataset, cell annotation assignment was based on the cell type with the highest proportion present in each cluster. Next, Celltypist[75] was used based on the *Immune_All_High* model to obtain another set of annotation independent of the cell annotation in the first step. This is then followed by manual annotation using signature genes of cell types from literatures and DEGs of each cell cluster, and a third set of annotation was manually performed. Based on the three sets of annotations, at the level of lymphoid/myeloid/non-immune cell type classifications were split into their corresponding lineage clusters. Further splitting of lymphoid cells into $CD4^+$T, $CD8^+$T, B and plasma cells, and other lymphoid cells were performed. After splitting, further clustering and DE analysis was done using the same procedures described above, to obtain a final set of cell type annotation at a finer granularity.

Comparison of cell type proportions

For each cell type, we performed two-proportions z-test between each comparing groups (e.g., R versus NR; Pre versus Post) to assess whether the proportion of each cell type were statistically different between the two groups. Fold change between the groups for each cell type was tabulated and multiple testing correction was done using BH FDR.

GSEA, GO, and KEGG pathway analyses

For each cell type, DE analysis was performed to obtain cell type-specific DEGs with multiple-testing correction using BH FDR as described above. Genes with FDR <0.05, were retained and subjected for GSEA analysis using the biological hallmark, regulatory target, oncogenic signature, immunologic signature gene sets obtained from the MSigDB[44] and fgsea R package. GO and KEGG pathway analyses were performed using limma v3.52.4.[76]

Trajectory and RNA velocity analysis

Pseudotime trajectory was performed using PC1 and monocle2[77] on post-treatment patients to observe post-therapeutic cell-state transitions in $CD4^+$ and $CD8^+$ T cell lineages. For both methods, cells were assigned with a pseudotime value based on PC1 or constructed graph in monocle2. For monocle2, naive cells from the CD4 or CD8 lineages were chosen as the origin of cell states for the two lineages, and trajectories started from the group of naive cells from which their occupancies in the trajectory graph nodes were the highest.

Gene regulatory network analysis

We performed GRN analysis of the cell types present across strata (i.e., Pre, Post, ICB-R, and ICB-NR) using SCENIC[39] based on their gene expression counts. GENIE3[78] was used for the network inference, based on trained random forest models. To identify genes which were active in the GRN, AUCell[39] was used to assess the activity of each regulon based on the area under the receiver operating characteristic curve (ROC) curve (AUC). T-test was performed to identify TFs which were significantly differentially expressed between response or treatment conditions across different cancer types based on the regulon activity scores. TFs with AUC >0.2 were retained for each cancer type and each stratum. Clustering analysis was performed to assess the level of similarities between different cancer types and strata based on their TF activity scores. Gene expressions of the selected TFs were also used to examine gene-expression level similarities.

Cell-cell communication

We decipher cell-cell interactions of the IRATs with other $CD4^+$/$CD8^+$ T cells in both R and NR groups based on the aggregated rankings of results from Natmi,[79] Connectome,[80] logFC Mean, SingleCellSignalR,[81] and CellphoneDB[41] using Liana.[82,83] Highly ranked ligand-receptor pairs were retained at aggregated rank significance of <0.01. We tabulated the number of these significant interactions separately for ligand and receptor interactions in each cell type and compared the interaction frequencies within each treatment and response conditions (i.e., Pre versus Post, and R versus NR). Ranked based on aggregated rankings, top ligand-receptor interactions were retained for further analysis.

Multimodal TCR analysis and the identification of response-specific TCRs

Using the data from Discovery Cohort 2, we examined single-cell immune profiling data across eight cancer types from 133 patients, resulting in 213 samples segmented into 73 pre-treatment and 140 post-treatment, the latter comprising 50 clinically responsive and 49 resistant cases, with the remaining presenting indeterminate response status. Discovery Cohort 2 also consisted of 27 normal tissue samples from 26 individuals as controls. Quality control efforts yielded 117,094 unique clonotypes from the cancer cohort and 29,817 from normal tissues.

For each cancer type, we processed their multimodal TCR single-cell immune profiling data using scRepertoire.[84] Samples with mapping reported defects were discarded. Here, we considered a TCR clonotype as the amino acid sequence of the CDR3 region of a cell. Clonotypes with only one of the TRC alpha or beta chains were discarded. TCR data were also collected from healthy or normal adjacent samples (Table S1) and filtered based on the sample procedure as the cancer TCR samples. In order to obtain response-specific TCRs, for each cancer, in the responder group, we compared the TCR repertoires present in the responder samples with the TCRs from the non-responder, treatment-naïve, and normal samples, and retained only the TCR sequences present uniquely in the responder samples, which gave us responder-specific TCR repertoires. Likewise, comparing TCRs from the responder, treatment-naïve, and normal samples, and retained only the TCR sequences present uniquely in the non-responder samples gave us non-responder-specific TCRs. For the cancer samples, we integrated the multi-modal TCR sequences with their corresponding scRNA-Seq data and assigned the cells with respective TCR information. Clonal frequencies for treatment-specific and response-specific TCR clones were computed for each cell type across cancer types. To compare clonal expansions between response groups, fisher exact test was used to assess the proportional difference between the groups and t-test was used to compare the clonal frequency distribution means between the groups for each cell type. Fold change between the R and NR groups for each cell type was tabulated and multiple testing correction was done using BH FDR.

Construction of response prediction model and response index (RI)

Using data from Discovery Cohort 1 and 2, response prediction model was based on ICB-response-specific TCR repertoires and enriched MSigDB[44] pathways of the IRATs, i.e., $CD4^+$ CXCL13+ Tfh, $CD4^+$ TGFβ1+ T, $CD8^+$ CXCL13+ T, and $CD8^+$ Temra. In detail, first, DE analysis was performed on post-ICB treated samples with ICB-response status, and independently for each cell type and each cancer type in either of the response groups, a two-tailed Wilcoxon Rank-Sum test was performed to compare each cell type from each treatment-response group and cancer type with all other groups to obtain a set of DEGs specific to each cell type, cancer type, and response status. Bonferrroni correction was done to correct for multiple testing. Genes with Bonferroni-adjusted $p < 0.05$ were retained as DEGs for each cell type group. For each DEGs set, genes were ranked based on decreasing average log2FC to construct a ranked gene-level statistics for conducting GSEA[85,86] by comparing with gene sets from H (hallmark gene sets), C2 (curated gene sets such as KEGG and Reactome pathways), and C6 (oncogenic signature gene sets) of the Human Collections in MSigDB.[44] Adaptive multilevel Monte Carlo approach[86] was used with 10,000 permutations in the enrichment analysis. For each cell type and cancer type, enriched pathways with $p < 0.05$ were compared between the responder and the non-responder groups, and pathways which were positively enriched (i.e., positive enrichment scores (ES)) in the responder group as compared to the non-responders were selected for the construction of GSEA index. For each of the IRATs in each cancer type, a corresponding GSEA index was constructed by summing up the ES scores of their selected pathways. These indices will be contributing to the data used for response prediction. Next, to obtain ICB-response-specific TCR repertoires in each cancer type, for each of the response group in each cancer type, which were made up of post-treatment samples, we compared the TCR repertoires in the group with the TCRs in the opposing-response group, and subsequently compared with the TCRs from the treatment-naïve group and healthy samples from the tissue sites corresponding to the cancer type and remove TCRs which were also found in these subgroups (Figure 4A).

For each cell type, a clonal FC index was then constructed by computing the log2FC of the proportion of unique ICB-responder-specific TCR repertoires in the ICB-response-specific TCR repertoires against the proportion of unique ICB-non-responder-specific TCR repertoires in the ICB-response-specific TCR repertoires (Figure 4B). These clonal FC indices, together with the GSEA indices, will be used as the dataset for ICB-response prediction.

Based on the information, we split 70% of the data into the training cohort and the remaining 30% into the testing cohort, and trained in a generalized linear model (GLM) with elastic net regularization and 10-fold cross validation (glmnet, alpha = 0.3),[87] with the data as observations, and the ICB-response status (i.e., 1 = Responder, 0 = Non-responder) as the response variable. Binomial was used to model the response variable distribution. The Lasso penalty accounts for 30% of the penalty and Ridge penalty accounts for the remaining 70%.[87] To avoid overfitting, the whole procedure undergone bootstrapping of 100 rounds with random reassignment of data into training and testing sets in each round, and within each round, 10-fold cross validation was used for the GLM model fitting. Mean AUC was tabulated by taking the average of the AUCs obtained from the 100 rounds of bootstrapping. To assess the weightage of the clonal FC indices and GSEA indices, coefficient of each index obtained from fitting the GLM model in each round was scaled with standard deviation and the standardized coefficients of each index from the 100 rounds were average to obtain its final average weightage (absolute weightage). Based on the predicted response classes based on the 100 sets of testing data after the bootstrapping procedure, for each sample, the number of times the sample was chosen in the test dataset across the 100 times of boostrapping procedure ranges from 21 to 43. Majority voting was to assign the final predicted response class for each sample based on the predictions obtained from the 100 rounds of bootstrapping procedure. For each sample, the final predicted class will be marked as 0 (Non-responder) if less than or equals to 50% of the predicted classes obtained from boostrapping equals to 1 (Responder), while the final predicted class will be assigned as 1 (Responder) if more than 50% of the predicted classes equals to 1. Confusion matrix and its corresponding statistics (accuracy, Kappa, sensitivity, etc.) were constructed using the caret R package (v6.0-94) and the final response class obtained based on majority voting (Figure 4F).

Finally, in order to obtain a single response cut-off value for each sample, we further combined the GSEA indices and clonal FC indices to obtain a final response index (RI). $CD4^{+}$ CXCL13+ Tfh and $CD8^{+}$ CXCL13 + T were first classified as the tumor-suppressive (TS) group, and $CD4^{+}$ TGFβ1+ T as well as $CD8^{+}$ CXCL13 + T were considered as the tumor-promoting (TP) group. For each sample, a composite GSEA index was tabulated by summing up the GSEA indices of the cell types from both TS and TP groups. This is followed by obtaining a composite clonal FC index by taking the absolute sum of the clonal FC indices of the cell types from the TS group, divided by the sum of clonal FC indices from the TP group. The final RI was computed by the product of the composite GSEA and composite clonal FC indices. RI was formulated as shown,

$$RI = \left| \frac{\sum_{k=1}^{N} \frac{F_R}{F_{NR}}}{\sum_{k=1}^{Q} \frac{F_R}{F_{NR}}} \right| \times \left(\sum_{k=1}^{N} \sum_{l=1}^{M_k} ES_{l,k} + \sum_{k=1}^{Q} \sum_{n=1}^{N_k} ES_{n,k} \right)$$

where F is the clonal frequency for the R or the NR group, and N and Q are the total number of cell types in the TS and TP groups respectively. M_k is the total number of signature gene sets in cell type k in the TS group, and N_k is the total number of signature gene sets in cell type n in the TP group (Figure 4G).

Survival analysis based on bulk RNA-Seq data

To validate the impact of the expression of the IRATs on patient survival after ICB treatment, four post-immunotherapy RNA-Seq dataset were used, including IMvigor210 cohort (348 UC patients)[45]; Liu2019 skin cutaneous melanoma (SKCM) dataset (121 patients)[47]; Riaz2017 SKCM dataset (50 patients)[21]; and Braun2020 ccRCC dataset (181 patients)[46] were used. Gene expression of cell-type-specific signature markers were used for separating the patients into high-expression and low-expression groups using the maximally selected rank statistics (MSRS),[48] for cutpoints between the 10% and 90% quantile of the mean gene expression of each marker using the upper bound of the p-value.[88] In cell-type-specific signature markers, we have in addition also looked at the expression ratio of CXCL13 and TGFβ1 and how a relative higher CXCL13 expression could impact on the survival. MSRS method was also used for splitting patients for the ratio of CXCL13/TGFβ1 into higher and lower ratio group. Tumor mutational burden (TMB) was utilized in combination with the gene expression groups and was grouped into high or low TMB based on MSRS method. Kaplan-Meier survival curves were plotted and the survival difference between the two groups were computed using log rank test. Cox proportional-hazards model was applied to test for the association of the expression of each signature marker on patient survival (Figure 5E).

QUANTIFICATION AND STATISTICAL ANALYSIS

Statistical calculations were performed using R. Statistical details for specific analysis are provided under each sub-section. p-values and FDR values <0.05 were considered in all analyses to be statistically significant.

Cell Reports Medicine, Volume *6*

Supplemental information

Deciphering immune predictors of immunotherapy response: A multiomics approach at the pan-cancer level

Xuexin Li, Lu Pan, Weiyuan Li, Bingyang Liu, Chunjie Xiao, Valerie Chew, Xuan Zhang, Wang Long, Florent Ginhoux, Joseph Loscalzo, Marcus Buggert, Xiaolu Zhang, Ren Sheng, and Zhenning Wang

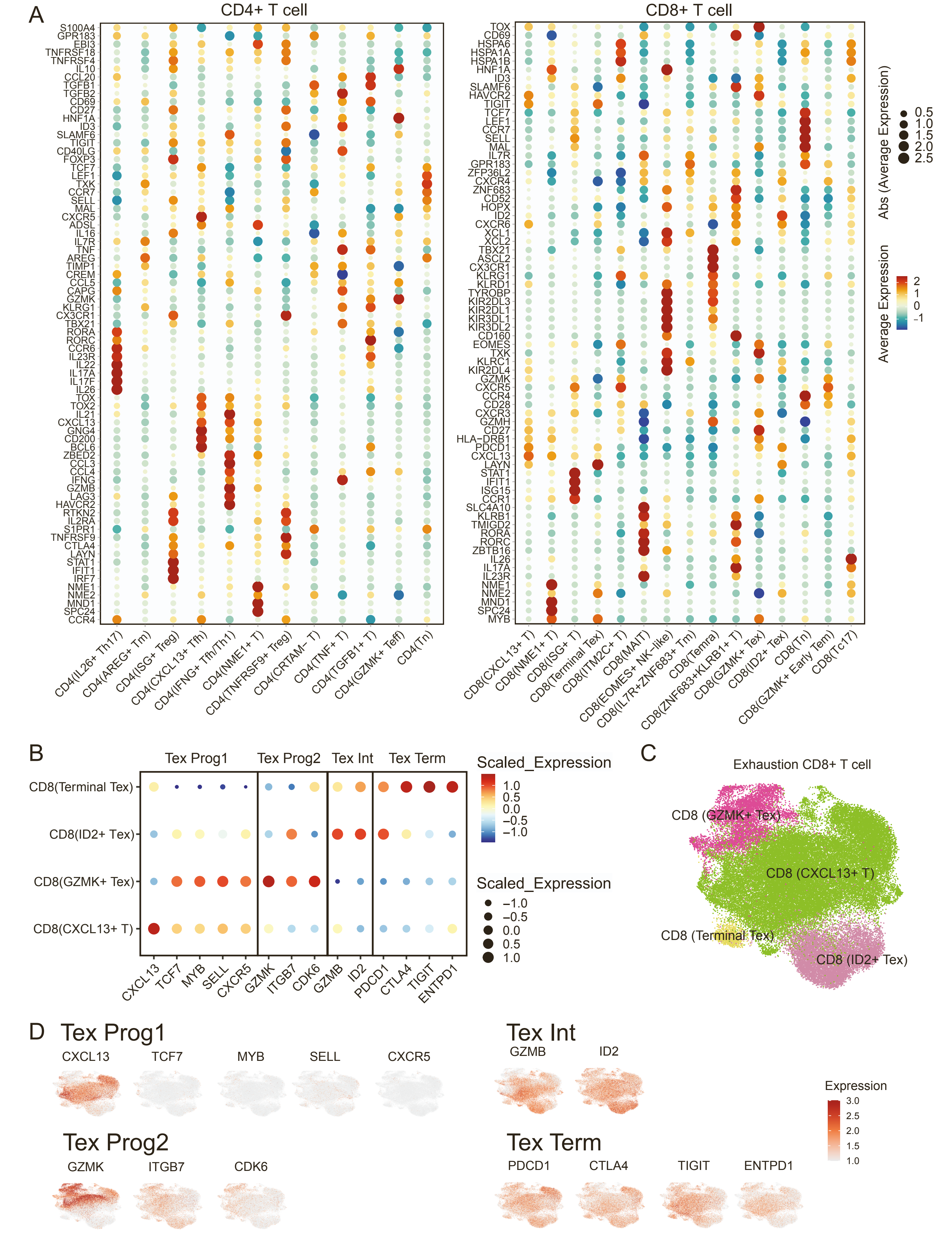

Figure S1. Expressions of signature genes in CD4 and CD8 T cells. (A) Signature genes for all the CD4 and CD8 T cells. Size of dots represented percentage of cells expressing the genes, and color gradient indicated average expression of the genes. (B) Signature genes for the four exhaustive CD8 T cell types. (C) UMAP representation of the four exhaustive CD8 T cell types and (D) the average gene expression of the signature genes across these cells. Related to Figure 2.

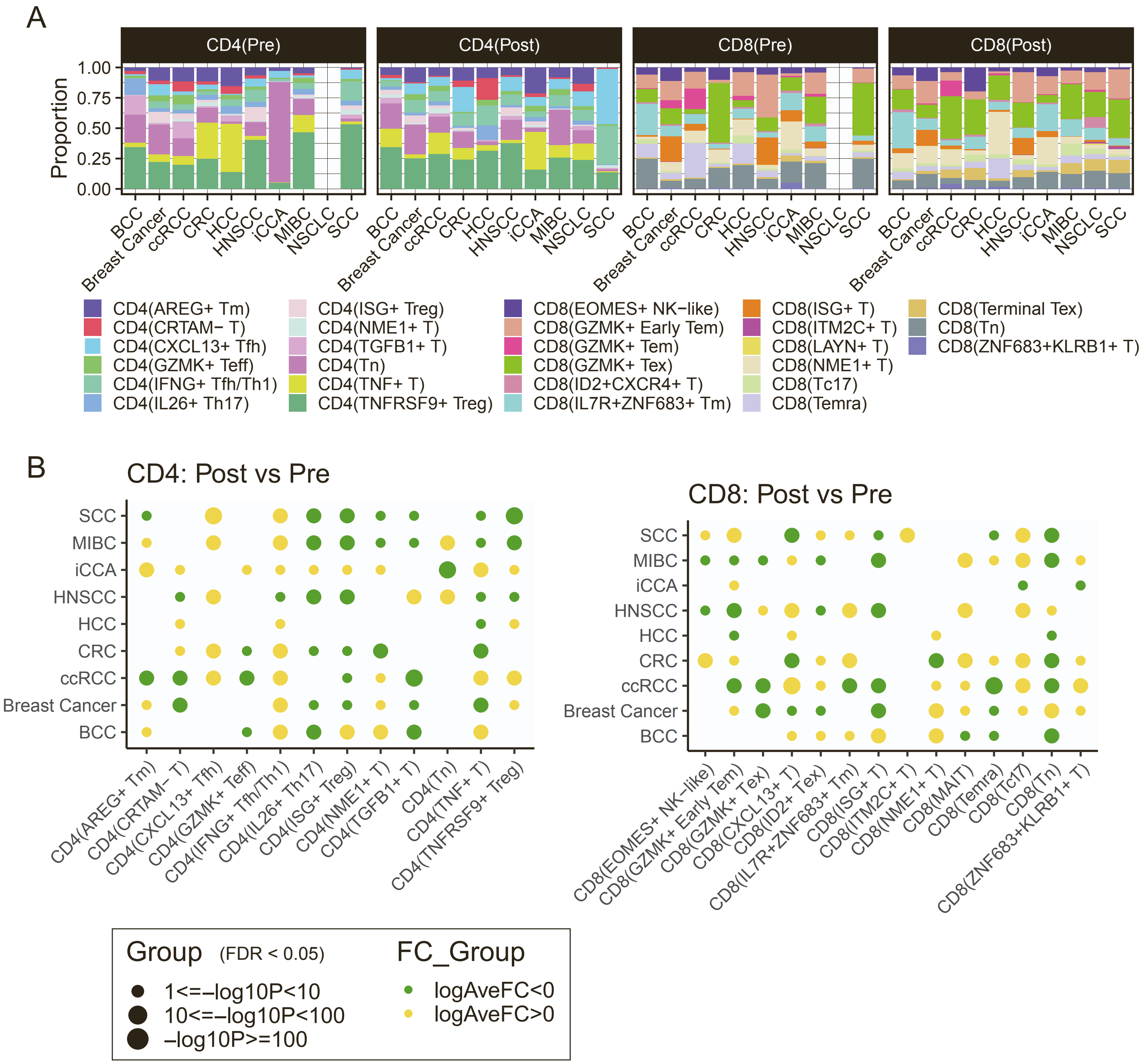

Figure S2. Phenotypic landscapes of treatment-naïve and post-treatment groups. (A) The proportion of cell types across cancer types in treatment-naïve and post-treatment groups. (B) Comparison of the proprtions between the treatment-naïve and post-treatment groups in both CD4/CD8 T cell. Two-proportions z-test was used to compare between the groups. Only comparisons with FDR < 0.05 were shown. Related to Figure 2.

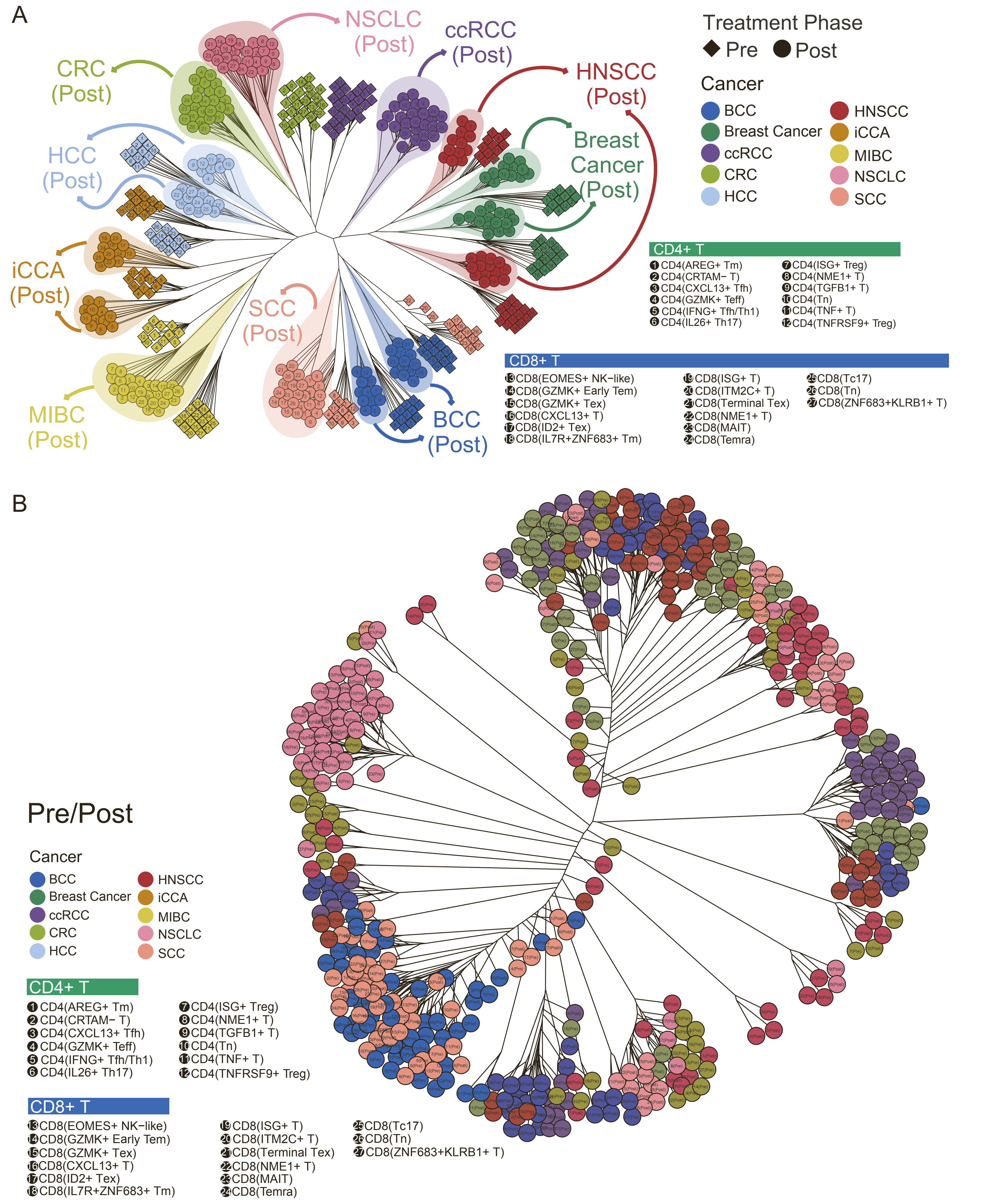

Figure S3. Clustering of TFs from the treatment-naïve and post-treatment groups across cancer types. Clustering of TFs based on their (A) transcriptional activities as well as (B) gene expressions across cancer types in the Pre and Post groups. Related to Figure 2.

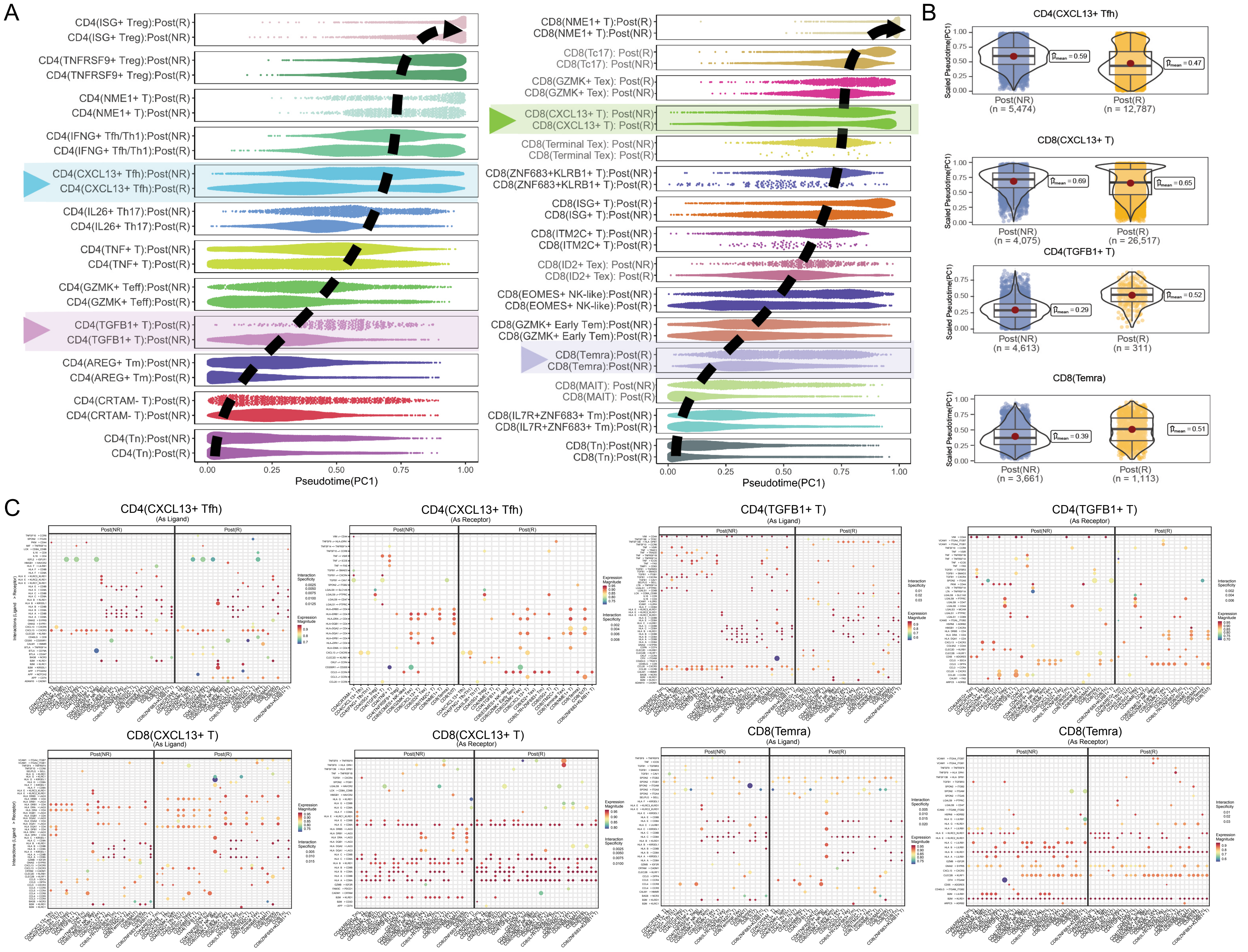

Figure S4. Pseudotime and cell to cell interaction. (A) Pseudotime analysis based on PC1 across all CD4 and CD8 T cells. (B) Box plots showing the result of t-test comparing the pseudotime between the response groups. (C) Top ligand-receptor binding interactions in the IRATs. Size of the dots represented interaction specificity and color gradient indicated the expression of the interaction pairs. Related to Figure 3.

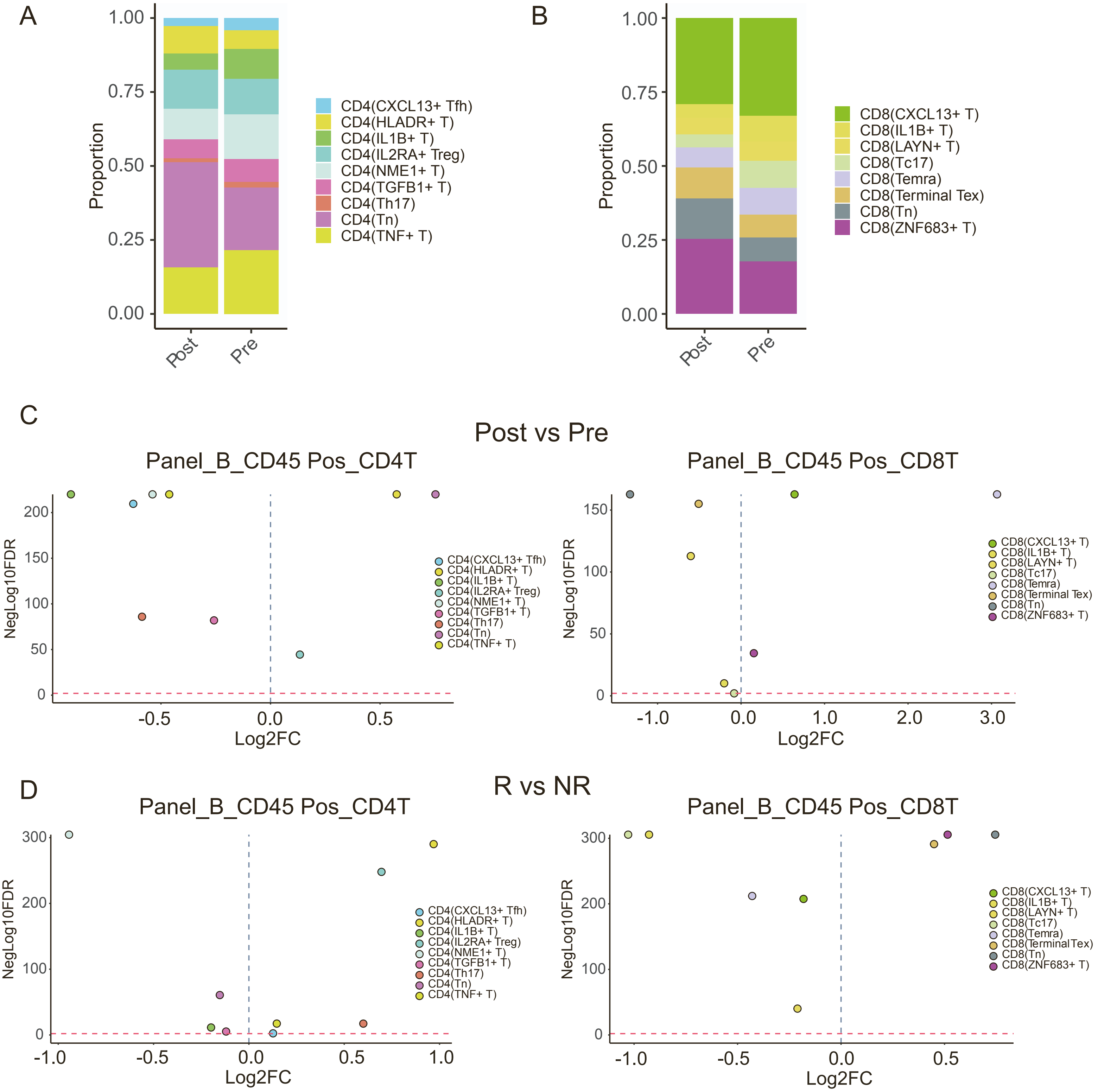

Figure S5. CyTOF proportion analysis result . (A) Proportion of CD4 cell types and (B) CD8 cell types in the treatment-naïve and post-treatment samples. (C) Result of two-samples proportion Z-test between the treatment groups and (D) response groups. Related to Figure 5.
